# Light control of three‐dimensional chromatin organization in soybean

**DOI:** 10.1111/pbi.14372

**Published:** 2024-05-19

**Authors:** Zhu Li, Linhua Sun, Xiao Xu, Yutong Liu, Hang He, Xing Wang Deng

**Affiliations:** ^1^ National Key Laboratory of Wheat Improvement Peking University Institute of Advanced Agricultural Sciences, Shandong Laboratory of Advanced Agricultural Sciences at Weifang Shandong China; ^2^ School of Plant Science and Food Security Tel Aviv University Tel Aviv Israel; ^3^ School of Advanced Agriculture Sciences and School of Life Sciences, State Key Laboratory of Protein and Plant Gene Research Peking University Beijing China

**Keywords:** Hi‐C, light response, chromatin organization, *SAURs*

## Abstract

Higher‐order chromatin structure is critical for regulation of gene expression. In plants, light profoundly affects the morphogenesis of emerging seedlings as well as global gene expression to ensure optimal adaptation to environmental conditions. However, the changes and functional significance of chromatin organization in response to light during seedling development are not well documented. We constructed Hi‐C contact maps for the cotyledon, apical hook and hypocotyl of soybean subjected to dark and light conditions. The resulting high‐resolution Hi‐C contact maps identified chromosome territories, A/B compartments, A/B sub‐compartments, TADs (Topologically Associated Domains) and chromatin loops in each organ. We observed increased chromatin compaction under light and we found that domains that switched from B sub‐compartments in darkness to A sub‐compartments under light contained genes that were activated during photomorphogenesis. At the local scale, we identified a group of TADs constructed by gene clusters consisting of different numbers of *Small Auxin‐Upregulated RNAs* (*SAURs*), which exhibited strict co‐expression in the hook and hypocotyl in response to light stimulation. In the hypocotyl, RNA polymerase II (RNAPII) regulated the transcription of a *SAURs* cluster under light via TAD condensation. Our results suggest that the 3D genome is involved in the regulation of light‐related gene expression in a tissue‐specific manner.

## Introduction

Investigation of epigenetic mechanisms, including DNA methylation, histone modifications and variants, regulatory non‐coding RNAs and even higher‐order chromatin structure, has expanded rapidly in recent decades (Berger *et al*., 2009; El‐Osta and Wolffe, 2001). Studies have established that structures created by wrapping of DNA around histones octamers further organize genetic information through formation of chromatin loops, TAD domains, A/B compartments and chromosome territories (CTs) (Kempfer and Pombo, 2020; Lieberman‐Aiden *et al*., 2009; Misteli, 2020). Understanding how chromosomes fold provides insights into the complex relationships between chromatin structure, gene activity and the functional state of the cell (Santos *et al*., 2020). The establishment of high‐resolution Hi‐C contact maps has revealed that hierarchical 3D genome organization is conserved but distinct between mammals and plants (Doğan and Liu, 2018; Moissiard *et al*., 2012; Rao *et al*., 2014). Hi‐C approaches have not only confirmed the presence of CTs in plants but have also revealed the compartmentalization of plant genomes into A and B compartments, which correspond to transcriptionally active regions marked by a high density of genes and active histone modifications and transcriptionally repressive regions enriched with DNA methylation, inactive histone modifications and transposable elements, respectively (Concia *et al*., 2020; Dong *et al*., 2017, 2018, 2020; Lieberman‐Aiden *et al*., 2009). At a mega‐base scale, canonical TADs in mammals are established and insulated from each other via chromatin loop extrusion mediated by the CTCF/cohesin complex (van Ruiten and Rowland, 2021; Xiang and Corces, 2021). However, the absence of CTCF proteins in plants suggests that establishment and maintainence of TADs in plants may be regulated by a mechanism distinct from that found in animals (Dixon *et al*., 2012; Domb *et al*., 2022; Feng *et al*., 2014; Heger *et al*., 2012; Nora *et al*., 2012). In addition to cohesin, RNA polymerase II (RNAPII) has also been hypothesized to act as a molecular motor involved in the dynamics of chromatin spatial organization (Hsieh *et al*., 2015; Rowley *et al*., 2017; Ulianov *et al*., 2016). Evidence in zygotes of *Drosophila melanogaster* indicated that TAD emergence coincides with transcriptional activation, the transcriptional activity of self‐interacting domains across eukaryotes, including *Saccharomyces cerevisiae*, *Caenorhabditis elegens* and *Arabidopsis thaliana*, is closely associated with chromatin contacts and explains the majority of chromatin partitioning (Hsieh *et al*., 2015; Rowley *et al*., 2017; Ulianov *et al*., 2016). RNAPII‐mediated transcriptionally active interacting domains containing genes that display relatively higher expression continue to be identified in plants (Jiang *et al*., 2020; Wang *et al*., 2015). Optimization of Hi‐C approaches has revealed and categorized various long‐range DNA–DNA interactions in animals and plants. For example, promoter‐promoter interactions mediated by RNAPII in plants establish complex spatial transcriptional units for regulating cooperative gene transcription in a manner similar to promoter‐centered RNAPII loops in human cells (Li *et al*., 2012). In *Arabidopsis*, RNAPII was figured out to tether on the genomic region of euchromatin and mediate with the transcriptional regulation (Deng *et al*., 2023; Grob *et al*., 2014; Sun *et al*., 2024). No matter which hierarchical scale the chromatin conformation is folded, the spatial structure of genome is closely correlated to the epigenetic states in plants (Domb *et al*., 2022). Generally, the H3K9me2 is enriched with the pericentromeric regions, while the H3K27me3 and H3K4me3 always mark the euchromatin among the genome of *Arabidopsis* and soybean cultivar and spatial connectivity of chromatin associated with epigenetic marks cooperatively facilitate transcription (Deng *et al*., 2023; Liu *et al*., 2016; Sun *et al*., 2024; Wang *et al*., 2015; Yang *et al*., 2022). Our previous study also revealed that H3K27me3‐associated chromatin loops are conserved in *Arabidopsis*, *Oryza sativa* and *Glycine max* (Huang *et al*., 2021; Nützmann *et al*., 2020; Sun *et al*., 2023).

Given the increasing requirement for food security and soil fertility, increasing attention has been given to soybean (*Glycine max*), the world's foremost oilseed and the primary source of protein. Innovative crop breeding approaches have focused on improving soybean yield, quality and disease resistance (Foyer, 2016; Lee *et al*., 2011). Several studies have described the 3D genome of soybean. Comparison of the high‐resolution Hi‐C contact maps of wild soybean, cultivated soybean and the common bean suggest that the Rabl‐like configuration identified in big‐genome crops is also found in legume species and the conserved 3D‐chromatin structures, including A/B compartments and TADs, are defined at the chromosomal level (Ni *et al*., 2023; Wang *et al*., 2021). Moreover, the long‐range chromatin loops were found to display active histone modifications that mediate expression of genes duplicated during polyploidization and subsequent diploidization (Wang *et al*., 2021). These findings suggest a role for higher‐order chromatin structures in the regulation of soybean genes.

Light is a powerful stimulus that regulates plant development. Light significantly impacts seedling morphology (Macháčková, 1994). Exploration of nuclear architecture dynamics triggered by light signals in *Arabidopsis* seedlings revealed that *chlorophyll a/b‐binding proteins* (*CABs*) are relocated from the nuclear interior to the periphery during photomorphogenesis, as *CABs* are transcriptionally activated by light (C.‐M. Feng *et al*., 2014). Furthermore, an increase in total nuclear surface area and repositioning of heterochromatin towards the chromocenters were observed as well (Bourbousse *et al*., 2015). Although previous reports have described rapid compaction of chromatin structure within the nucleus in response to light as well as a reversible decrease in this large‐scale chromatin compaction with decreasing light intensity, our understanding of how this dynamic higher‐order chromatin organization contributes to light‐regulated gene expression is critical but incomplete (van Zanten, Tessadori, Bossen, *et al*., 2010; van Zanten, Tessadori, McLoughlin, *et al*., 2010).

To enhance our understanding of the biological function of the 3D genome in soybean, we investigated hierarchical chromatin architectures in the cotyledon, apical hook and hypocotyl by Hi‐C. We not only identified A/B compartments, A/B sub‐compartments, TADs and long‐range chromatin interactions in each organ but also found that chromosome‐level changes in chromatin organization induced by light differed between the three tissues. By integrating 3D genomics, epigenomics and transcriptomics, we discovered a group of TADs composed of clustered *Small Auxin‐Upregulated RNAs* (*SAURs*). Sixty‐seven percent of all *SAURs* were found to be clustered and these clustered *SAURs* displayed tissue‐specific regulation in response to light and co‐expression in the apical hook and hypocotyl. In addition, the TAD constructed by a *SAURs* cluster mediated RNAPII regulation of *SAURs* by compacting chromatin in response to light. Our findings, along with other available data, reveal the existence of chromatin domains comprised of light‐associated genes whose expression is mediated by RNAPII in soybean.

## Results

### Genome‐wide chromatin organization dynamics are influenced by light

**Figure 1:**
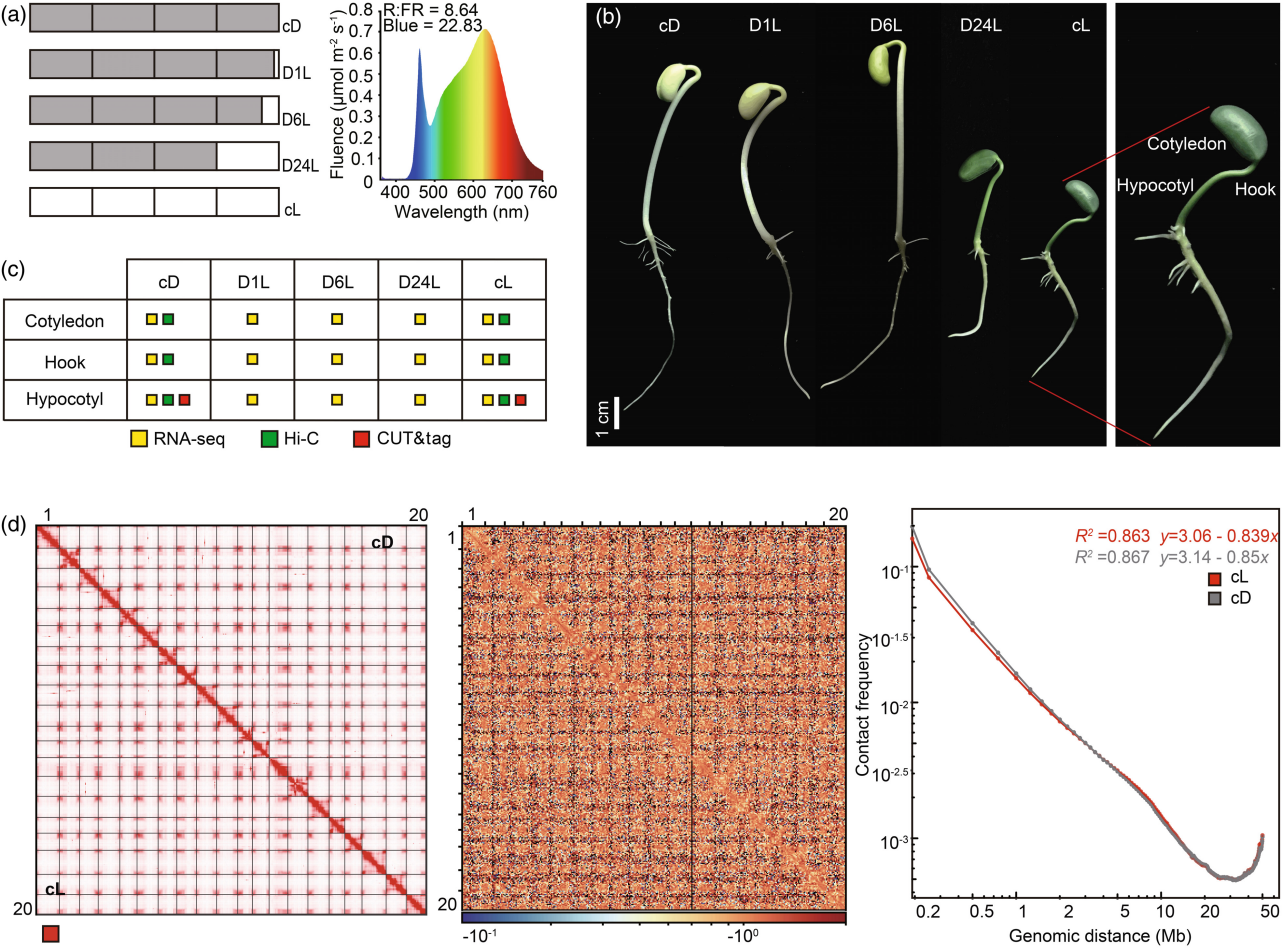
Light induces chromatin rearrangements at the chromosome level. (a) Left: Approach designed to observe photomorphogenesis in soybean, involving five growth conditions: continuous darkness (cD), continuous white light (cL), or transferred from darkness to light and then kept in white light for 1 h (D1L), 6 h (D6L) and 24 h (D24L). Grey: dark treatment. White: white light treatment. Right: Light spectral compositions of white light. (b) Morphological comparison of 4‐d‐old Wm82 soybean grown in cD, D1L, D6L, D24L and cL. Scale bar corresponds to 1 cm. (c) Three genomic approaches (RNA‐seq, Hi‐C and CUT&tag) employed to study the cotyledon, apical hook and hypocotyl. CUT&tag: Cleavage Under Targets and Tagmentation (CUT&tag) for H3K9me2, RNAPII Ser2P, H3K4me3, H3K27ac and H3K27me3. Hi‐C: *in situ* Hi‐C experiment against the cotyledon, apical hook and hypocotyl of cD and cL. (d) The genome‐wide chromatin architecture of hypocotyl described using chromatin interaction frequency at 100‐kb resolution under cD and cL conditions. Left: The genome‐wide Hi‐C interaction heatmap calculated by comparing cD and cL conditions. Diagonal line values are set to 0. Middle: Differential contacts were generated from the contacts remaining after excluding the contacts from the cD treatment from the those from the cL treatment, shown as a log_2_ratio matrix. Right: The averaged scaling plot of contact probabilities over increasing genomic distances for all soybean chromosomes. Genomic bin size is 100 kb. Grey: constant darkness. Red: constant light.

To investigate the 3D structure of the soybean genome and its dynamics during seedling establishment under light, we designed an approach to study photomorphogenesis in germinating soybean seedlings. Treatments consisted of constant dark, dark followed by 1, 6 and 24 h of light and constant white light (Figure 1a). A previous study demonstrated that *Arabidopsis* seedlings switching from skotomorphogenesis to photomorphogenesis display a de‐etiolated phenotype in a light‐dependent manner (Kempfer and Pombo, 2020). We observed comparable phenomena during seedling development following light stimulus in soybean. Specifically, seedlings germinating under constant dark exhibited elongated hypocotyls, unexpanded apical hooks and yellow cotyledons, which differed from the phenotype observed in seedlings grown in constant light, which displayed green aerial parts and open apical hooks (Figure 1b). During establishment of photomorphogenesis, seedling light perception was measured by recording the appearance after 1, 6 and 24 h of light stimuli (Figure 1b). We found no clear difference in the phenotypes between seedlings after 1 h of light stimuli and those growing under constant darkness. We observed open apical hooks and greening cotyledons after 6 h of light and continuously extended apical hooks in de‐etiolated seedlings after 24 h (Figure 1b).

To investigate the role of chromatin structure in the regulation of light‐responsive genes during photomorphogenesis, we collected the cotyledon, apical hook and hypocotyl tissue from 4‐day‐old seedlings subjected to the five light conditions and analysed them by RNA‐seq. We also performed *in situ* Hi‐C with two biological replicates of each of the three organs treated with either constant darkness or constant light (Figure 1c). The similarity between Hi‐C interaction matrices evaluated by the Stratum‐adjusted Correlation Coefficient (SCC) showed the high quality of Hi‐C library (Figure S2a). HiC‐Pro quality control results indicated that the proportion of *cis*‐interaction reads in each replicate was higher than that of trans‐interactions; thus, all libraries were considered high quality. After merging the sequencing reads of the two biological replicates for each of the dark and light‐treated organ samples, we found that the average number of valid interaction reads was ~523 million per sample, which was approximately equal to that observed in previous studies (Ni *et al*., 2023; Wang *et al*., 2021) (Table S1). Additionally, we performed Cleavage Under Targets and Tagmentation (CUT&tag) of H3K9me2, RNAPII Ser2P (phosphorylation of serine 2 on the carboxy‐terminal domain of RNA polymerase II) (Buratowski, 2009; Hajheidari *et al*., 2013) and H3K27me3 on the hypocotyl after treatment with constant darkness or light. H3K4me3 and H3K27ac CUT&tag were conducted for the hypocotyl under constant darkness. The quality control of CUT&tag datasets is displayed in Table S2.

In eukaryotes, each chromosome is compacted separately into chromosome territories (Lieberman‐Aiden *et al*., 2009; Misteli, 2020; Santos *et al*., 2020). Thus, we first investigated whether our data described this conserved feature of genome organization. The Hi‐C contact map was generated from two biological replicates each for the cotyledon, apical hook and hypocotyl sampled after dark and light treatment (Figure 1d left; Figure S1 left). The resulting heatmap revealed chromosome territories in the soybean genome consistent with previously reported results (Ni *et al*., 2023; Wang *et al*., 2021). The Hi‐C data also showed that the general patterns of chromatin interaction in darkness and light displayed similar patterns among the three organs; chromatin contact density was strongly concentrated along the main diagonals and decreased with increasing linear genomic distance (Figure 1d left; Figure S1 left).

To assess the effect of light on the chromatin architecture in different organs, we constructed a differential heatmap where chromatin contacts common between darkness and light were removed; this analysis revealed a greater number of genome‐wide chromatin interactions under light conditions across the three sampled tissues (Figure 1d middle; Figure S1 middle). We then calculated the average intrachromosomal contact probability to quantitatively describe chromatin interactions between any two loci separated by a genomic distance, at the chromosome level. The resulting interaction decay exponents (IDEs) for the cotyledon, apical hook and hypocotyl in darkness and light were −0.865 and −0.877, −0.879 and −0.855, −0.850 and −0.839, respectively (Figure 1d left; Figure S1 right). These negative IDEs, the genome‐wide contact map and the differential contact map together indicated that, during both darkness and light, chromatin interactions declined with increasing genomic distance, until the genomic distance reached the telomere region, whose chromatin interaction strength monotonically increased with increasing genomic distance. Our data also showed that the chromatin contacts differentially formed during dark and light were distinct in the three organs (Figure 1d; Figure S1), suggesting that light signals directly trigger condensation of chromatin at the chromosome level in an organ‐specific manner.

Taken together, our data reveal that the soybean genome displays remarkable structural plasticity when shifting from etiolation to de‐etiolation during photomorphogenesis. By comparing Hi‐C heatmaps generated from seedlings subjected to continuous light and dark, we identified alterations in global chromosome conformation induced by light as well as differences in these light‐responsive changes between the cotyledon, apical hook and hypocotyl. These results demonstrate that 3D genome organization can differ between cells from different plant organs and can undergo organ‐specific rearrangements in response to light.

### Sub‐compartment patterning reflects the transcription landscape

**Figure 2:**
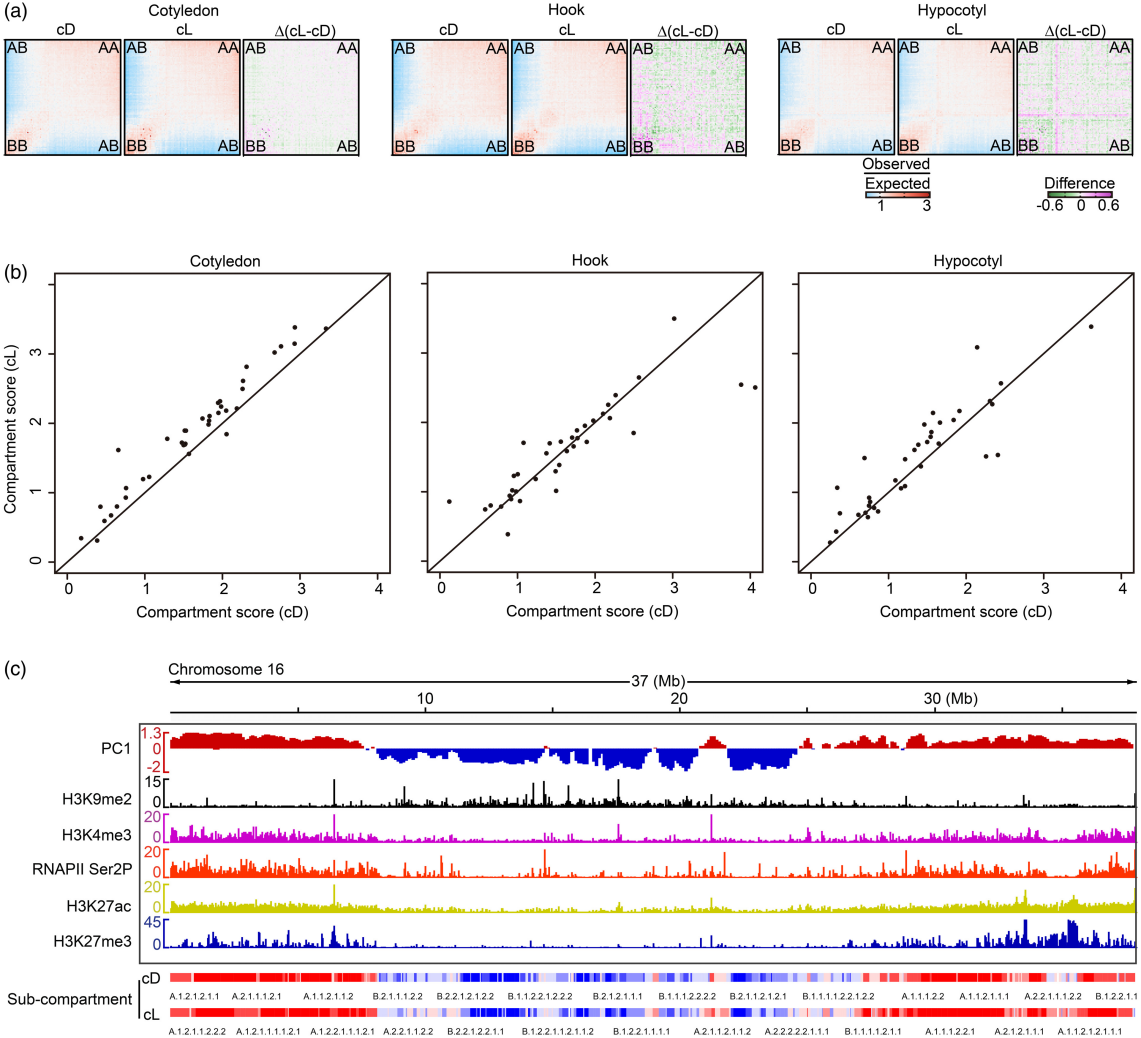
Euchromatin and heterochromatin regions were identified in soybean chromosomes. (a) The saddles (left, middle) and differential saddles (right) of chromatin compartmentalization comparing the *cis* chromatin interactions within a chromosome arm identified under cD and cL. cD: constant darkness. cL: constant light. Chromosome arms were divided into quantiles based on observed versus expected compartment scores between 100‐kb bins. The upper right and lower left show the interactions within A compartments and B compartments, individually, while the interactions between A and B compartments is performed on upper left and lower right. Differential saddle (right) with purple denoting more interactions in the sample in cL compared to the samples under cD. (b) Compartment strengths of 20 chromosomes from tissues treated under cD and cL. Compartment strength score was calculated from the number of A‐A and B‐B interactions divided by the A‐B interactions. (c) Sub‐compartment distribution of chromosome 16 from hypocotyl treated with constant dark. 1st track: A/B compartments under light are indicated by a positive PC1 (red) and a negative PC1 (blue), separately. 2nd‐6th tracks: the enrichment peaks for H3K9me2, H3K4me3, RNAPII Ser2P, H3K27ac and H3K27me3 in hypocotyl under cD were produced by CUT&tag. 7th and 8th track: The landscapes of A and B sub‐compartments during cD and cL over the whole chromosome are shown as red and blue bars, respectively.

Spatial chromosome compartmentalization is conserved but distinct among animals and plants. Several studies have suggested that each chromosome can be broken down into two compartments: active euchromatin labelled as the A compartment and inactive heterochromatin labelled as the B compartment (Dong *et al*., 2017). We identified both euchromatin A compartments and heterochromatin B compartments using principal component analysis (PCA) of our Hi‐C data. The chromatin interactions within the A and B compartments were stronger than chromatin interactions between the A and B compartments, as shown using a saddle plot (Figure 2a). Other studies have revealed that changes in nuclear architecture in response to light stimuli underlie reorganization of heterochromatin (Berger *et al*., 2009; Bourbousse *et al*., 2015; Dong *et al*., 2020; Rao *et al*., 2014). In our experiments, we found that in all three tissues, interactions in the B compartment were increased in light conditions compared to dark (Figure 2a), which resulted in increasing chromatin interactions of the whole chromosome arm, as shown in the scatterplot in Figure 2b. These increased chromatin interactions identified under light were closely correlated with the light‐induced compaction of chromatin interactions identified in the Hi‐C contact map (Figure 1d middle; Figure S1 middle).

**Figure 3:**
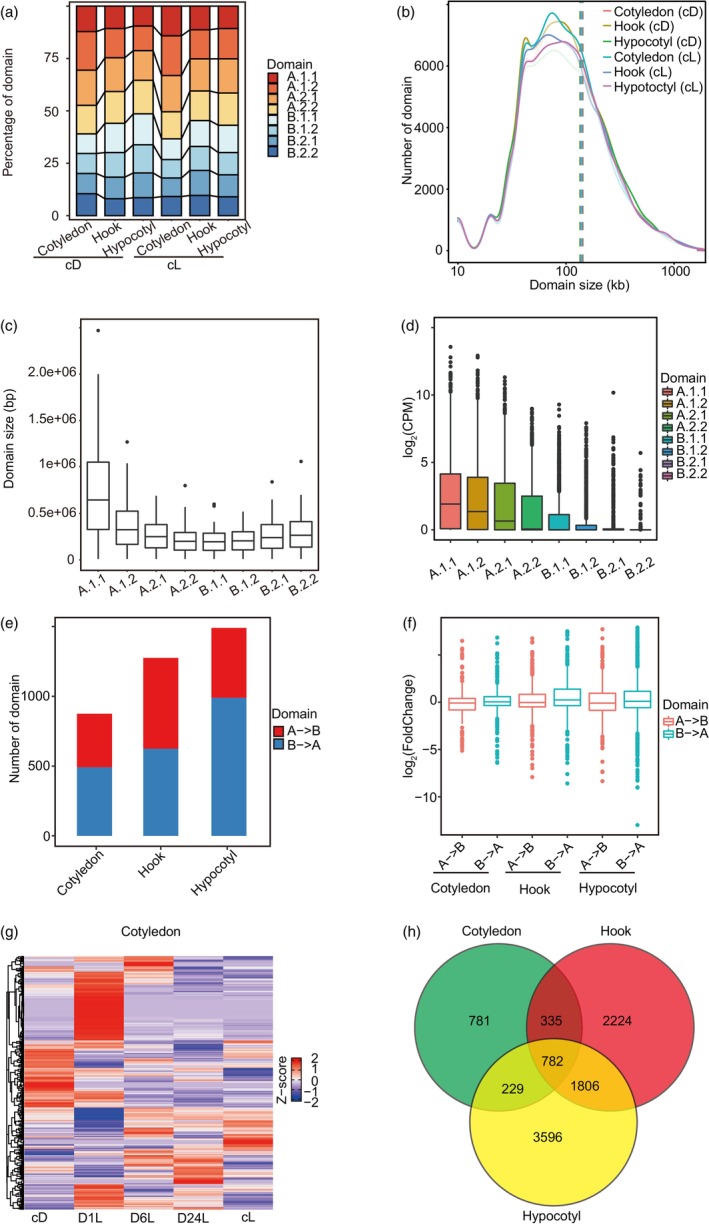
Ranking of A/B sub‐compartments is related to gene expression activity in the soybean genome across three tissues. (a) Proportions of each of the eight sub‐compartments (A.1.1, A.1.2, A.2.1, A.2.2, B.1.1, B.1.2, B.2.1, B.2.2) identified in cotyledon, apical hook and hypocotyl subjected to cD and cL. cD: constant darkness. cL: constant light. (b) Count distribution of all sub‐compartments (A.1.1, A.1.2, A.2.1, A.2.2, B.1.1, B.1.2, B.2.1, B.2.2) in dark‐treated cotyledon. (c) Domain size distribution of all types of sub‐compartments in cotyledon subjected to cD. (d) Transcriptional activity of each type of sub‐compartment in dark‐treated cotyledon. Gene expression is shown as log_2_CPM. (e) The proportion of dynamic sub‐compartments identified during the dark‐to‐light transition in each organ. B‐ > A: a group of B sub‐compartments in cD that switched to the A sub‐compartments following illumination. A‐ > B: a group of A sub‐domains in cD that switched to the A sub‐domains following illumination. (f) The expression level of genes within the dynamic sub‐compartments across the three tissues. Relative expression levels were calculated as log_2_foldchange. (g) The expression level of genes inside the domains that switched from B to A. The expression value of each light‐treated sample was normalized to the expression level of the dark‐grown sample. The relative expression levels (*Z*‐scores) were computed on a gene‐by‐gene (row‐by‐row) basis by subtracting the mean and then dividing by the standard deviation. The *Z*‐scores range from −2 to 2. (h) Venn diagram showing the overlap of upregulated genes induced by light in each tissue. Relative expression is presented as log_2_foldchange.

PCA is not powerful enough to annotate genomic regions with epigenetic features at high resolution as shown in Figure 2c, although the heterochromatin, occupying 57% of each chromosome (Schmutz *et al*., 2010) and the euchromatin were individually indicated by the negative and positive PC. Other approaches are required to further assess the diversity of chromatin states (Dong *et al*., 2018; van Ruiten and Rowland, 2021). To further explore the biological properties of genomic regions of interest in soybeans under dark and light conditions, we next further defined the sub‐compartmentalization of each chromosome using the Calder algorithm (Liu *et al*., 2021). Based on our high‐resolution Hi‐C map, we hypothesized that nested sub‐domains could be progressively clustered within each compartment (A/B compartments) and the chromatin activity of the subdivisions could be effectively measured by integrating CUT&tag data (H3K9me2, H3K4me3, RNAPII Ser2P, H3K27ac and H3K27me3) with Hi‐C data. The CUT&tag datasets were evaluated by Pearson's correlation coefficient analysis, which indicates good reproducibility of every two biological replicates in each dataset (Figure S2b). From this approach, we identified eight sub‐compartments, including four types of A sub‐domains (A.1.1, A.1.2, A.2.1 and A.2.2) inside the A compartment and four types of B sub‐domains (B.1.1, B.1.2, B.2.1 and B.2.2) within the B compartment, in each tissue under darkness and light (Figures 2c and 3a). Using chromosome 16 from the hypocotyl in darkness as an example, we found that A and B sub‐domains generally clustered at euchromatin regions (A compartments) and pericentromeric heterochromatin regions (B compartments), respectively, which is supported by the observed dense decoration with H3K4me3, RNAPII Ser2P, H3K27ac and H3K27me3 on euchromatin and the pericentromeric modification, H3K9me2, on highly repetitive sequence regions (Figure 2c). We found that the landscape of the sub‐domains in darkness was distinct from that observed under light (Figure 2c). And for responding to light, the deposition of histone marks, like H3K9me2 and H3K27me3, were changed (Figure S2c). From the above findings, we concluded that the chromatin states signified by these sub‐compartments were associated with the distribution of positive and negative histone modifications and that the defined sub‐compartments were more powerful for describing differences in chromatin state between light conditions in each organ than the larger two compartments.

A previous study reported that active A compartments typically feature high transcription activity and greater gene density (Xiang and Corces, 2021). In order to investigate the association between sub‐compartment domains and transcriptional activity, we measured transcription within the eight sub‐compartments using RNA‐seq. Quality control using PCA analysis showed a difference between treatments and satisfactory reproducibility between the two biological replicates used for each treatment (Figure S2d). The identified A and B sub‐domains were most commonly 80–90 kb in size (Figure 3b). Larger domains were mainly found in sub‐compartment A.1.1 (Figure 3c; Figure S3a) and we observed a higher proportion of A sub‐domains in the cotyledon compared to the apical hook and hypocotyl (Figure 3a). Interestingly, we found that the transcriptional activity of the A sub‐compartments was significantly greater than that of the B sub‐compartments and the level of transcriptional activity of each sub‐domain gradually decreased in order from A.1.1 to B.2.2; these results were shared among all tissue samples (Figure 3d; Figure S3b). Consistent with this finding, sub‐compartment assignments (from A.1.1 to B.2.2) of the pepper genome also correspond to the levels of gene expression (Liao *et al*., 2022). Interestingly, we observed sub‐compartment switching following illumination (A‐to‐B and B‐to‐A). This switch was observed during the transition from dark to light among all three tissues, but the apical hook and hypocotyl displayed a greater number of switching events than the cotyledon (Figure 3e). The genes within the domains undergoing the B‐to‐A shift exhibited higher expression than genes in the domains undergoing the A‐to‐B transition (Figure 3f). Gene expression heatmaps of the three tissues indicated that most genes were constantly activated during photomorphogenesis (Figure 3g; Figure S3c). Light‐activated genes inside sub‐domains undergoing the B‐to‐A transition showed high tissue‐specific expression (Figure 3h). Downstream Gene Ontology (GO) analysis for three tissues revealed that hypocotyl‐specific activated genes within B‐to‐A sub‐domains were enriched for GO terms related to light reaction, whereas genes upregulated genes in the apical hook were enriched for GO terms related to developmental processes (Tables [Link], [Link], [Link]).

In summary, we defined eight types of genomic sub‐compartments across three tissues in soybean; genome segments contained in these sub‐compartments displayed distinct histone modifications. We also found that the transcriptional landscape of these tissues was closely correlated with sub‐compartment patterning. Importantly, the repositioning of the sub‐compartments induced by light was related to the alteration of gene expression.

### TADs are present in soybean cotyledon, apical hook and hypocotyl

**Figure 4:**
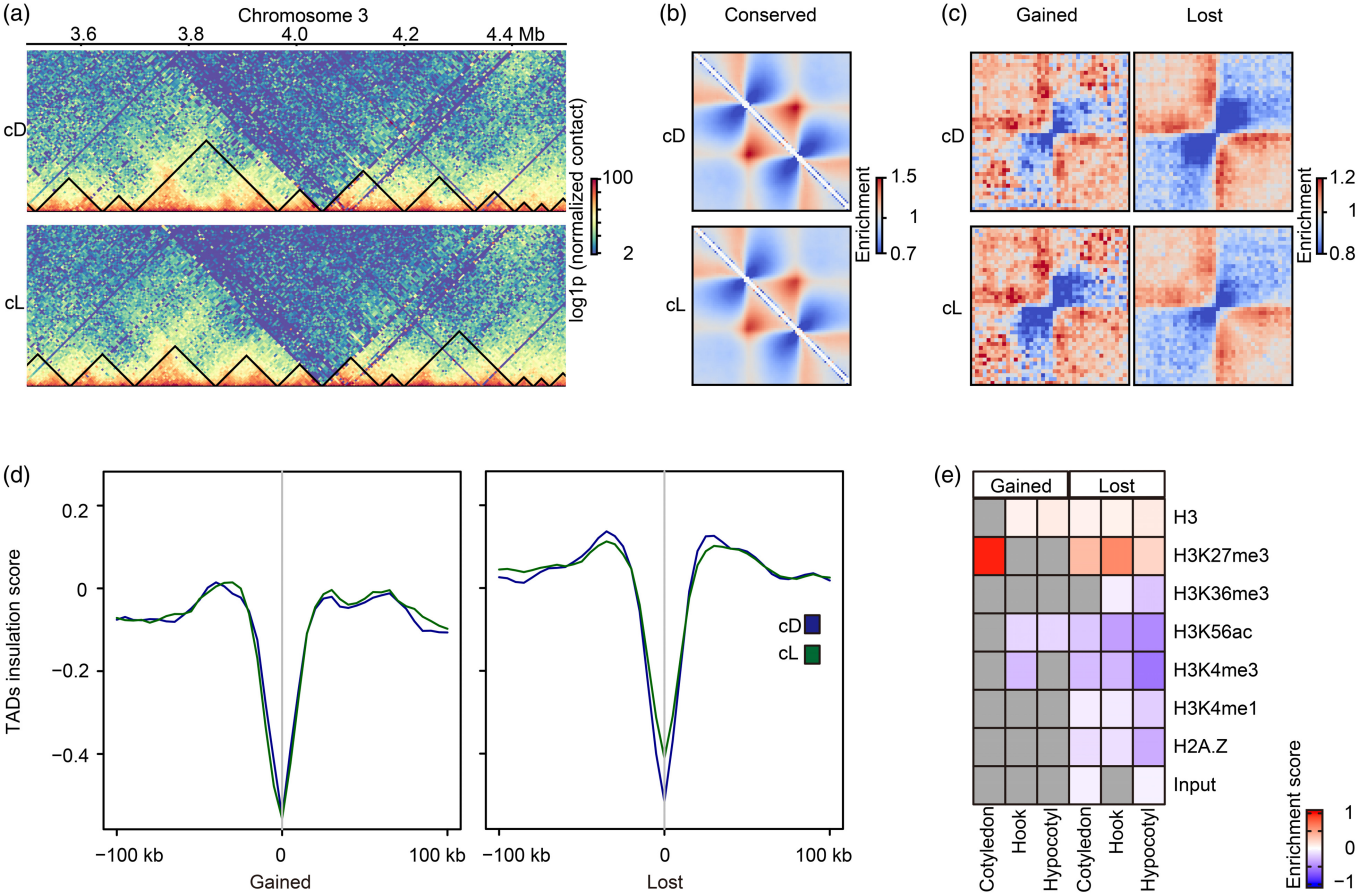
TAD dynamics correlate with H3K27me3 across three soybean tissues. (a) Shape of the TAD structure over a 1‐Mb region (3.5–4.5 Mb) on chromosome 3 shown in a snapshot of the Hi‐C heatmap under cD and cL. cD: constant darkness. cL: constant light. A matrix with a 5‐kb resolution was used for TAD calling. The normalized heatmap was transformed by log1p. (b) Aggregate TAD analysis (ATA) of conserved TADs in the cotyledon subjected to complete darkness and light. Five‐kb‐resolution Hi‐C matrices were used for pileups analysis of the TADs. Enrichment bar: average insulation values relative to random background, shown from the highest to lowest. (c) Aggregate TAD analysis (ATA) for the boundaries of dynamic TADs in the cotyledon. Gained TADs and lost TADs were separately defined as those with higher and lower insulation scores under cL compared to those under cD. Five‐kb‐resolution Hi‐C matrices were used for pileups analysis of the TADs. Enrichment bar: average insulation values relative to random background, shown from the highest to lowest. Average insulation was quantified by dividing the signal into two red squares (top left and bottom right corners) by the signal in the blue squares (top right and bottom left corners). (d) Profiling of the TAD insulation scores across the dynamic borders in the cotyledon. Blue: dynamic TADs under constant dark. Green: dynamic TADs under constant light. Gained TADs and lost TADs were separately defined as those with higher and lower insulation scores under light compared to those under dark. The borders of gained and lost domains are aligned and marked as ‘0’. (e) The heatmap of histone marks covering gained and lost TADs among the three tissues. Red and blue indicate the enrichment and depletion of histone marks, respectively, in dynamic TADs across the three tissues. Grey indicates that the epigenetic feature is neither enriched nor depleted (two‐sided Mannn–Whitney *U*‐test, *P* > 0.05). Relative density ranges from −1 to 1.

Given that other large plant genomes, such as those of wheat, maize and rice, exhibit many TADs, we hypothesized that TADs may also be found in the soybean genome (Dong *et al*., 2017; Xiang and Corces, 2021). To test this hypothesis, we used hicFindTAD to explore *cis*‐interactions in the Hi‐C matrix at 5‐kb resolution. Individual TADs were characterized as an insulated triangle domain with two boundaries along the diagonal of the Hi‐C matrix. We identified an average of 11 066 TADs with sizes ranging from 15 kb to 60 kb in each organ following exposure to dark and light (Figure 4a; Figure S4a). By quantifying the average strength of the TAD boundaries for each tissue under darkness and light, we found that the boundary strengths of some domains (the cotyledon: 3 399, the hook: 2 204, the hypocotyl: 2 204) were not different between darkness and light and we observed no changes in the domain location between conditions (Figure 4b; Figure S4b). The insulation score of corresponding domains was highly similar between dark and light and we defined these domains as conserved TADs (Figure S4c). However, in each soybean tissue investigated, a proportion of domains (the cotyledon: 91, the hook: 147, the hypocotyl: 87) were formed upon light stimuli, as indicated by increasing the average strength of domain boundaries under light (Figure 4d left), while another group of domains (the cotyledon: 320, the hook: 219, the hypocotyl: 582) was lost in a light‐dependent manner, as indicated by a decreased strength score after illumination. Increasing and decreasing TAD insulation scores, respectively, for the two groups of TADs also supported these observations (Figure 4d right; Figure S4e). We collectively designated these as dynamic light‐dependent TADs (Figure S4d). A recent study reported that the soybean genome regions comprising TADs are marked by higher amounts of repressive histone modifications (e.g., H3K27me3) compared to active epigenetic marks (e.g., H3K27ac) (S. Feng *et al*., 2014). We evaluated histone modifications throughout the identified light‐dependent TADs and found that the genomic regions that comprise these domains indeed contain abundant H3K27me3 compared to other modifications (Figure 4e). Our findings suggest that light triggers different mechanisms related to the formation and maintenance of TADs in different organs. Importantly, GO analysis of the genes inside these dynamic structures revealed a large proportion of genes involved in multiple biological processes. Within the TADs formed in the cotyledon genome upon light stimulation, we identified an enrichment for genes involved in symbiotic interactions (Figure S4f). Our results demonstrate that light modulates TAD dynamics and that epigenetic marks and encoded proteins may be involved in changes in TADs induced by light in an organ‐specific manner.

### TADs are constructed upon activation of the *SAURs* cluster under light via RNA polymerase II

**Figure 5:**
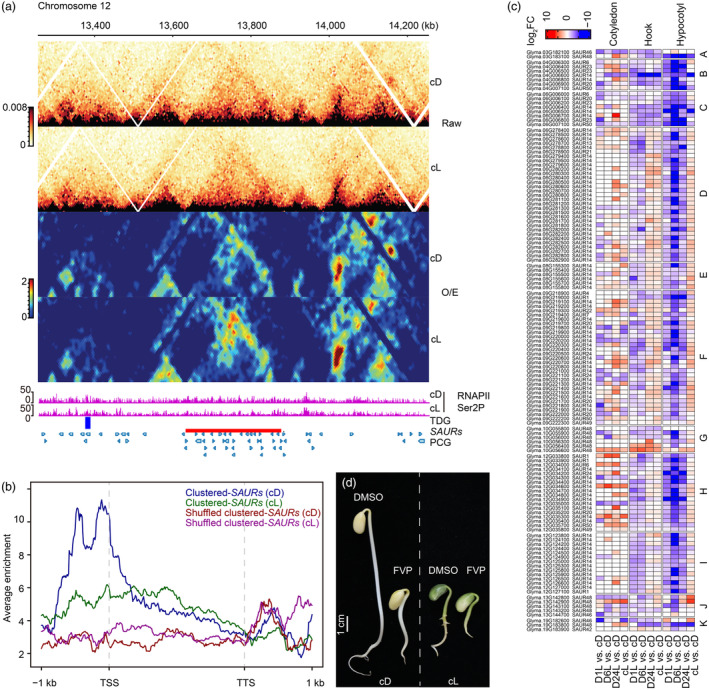
TADs comprised of *SAUR*s clusters differ in darkness and light. (a) Snapshot of the TAD identified at 13300–14250 kb on chromosome 12 in the hypocotyl under cD and cL. cD: constant darkness. cL: constant light. A matrix at 5‐kb resolution was used for TAD calling. Top panels show the raw heatmaps of 1‐kb Knight and Ruiz (KR)‐normalized contact frequencies under darkness and light. Below are heatmaps showing observed/expected (O/E) contact frequencies at 1‐kb resolution under darkness and light. The landscape of RNAPII Ser2P in cD and cL conditions is shown under the heatmap. TDG (blue bar): Tandemly Duplicated Gene. *SAURs* (red bar): Small Auxin‐Upregulated genes. PCGs (light blue bar): Protein‐coding genes. (b) Distribution of RNAPII Ser2P across the gene body of 25 *SAURs* (within chromosome 12:13 634 172‐13 876 042 bp). The average enrichment of RNAPII Ser2P over the ‘clustered *SAURs*’ under darkness and light is indicated by blue and green curves, respectively. Red and pink curves in the plots indicate background signal, which was calculated by distributing the clustered *SAURs* randomly throughout the genome for both cD and cL conditions. The flanking regions at the left side of the transcription start site (TSS) and at the right side of the transcription terminal site (TTS) are 1 kb in length. (c) The expression of clustered and non‐clustered *SAURs* during photomorphogenesis. Gene expression is represented by log_2_foldchange ranging from −10 to 10. A‐K indicate groups in which *SAUR* genes cluster by expression patterns under the five conditions. The names on the left of the heatmap are the soybean *SAURs* gene IDs followed by the name of the corresponding *Arabidopsis* homologues. (d) Morphological comparison of 4‐d‐old Wm82 soybeans treated with 15 μM FVP in cD and cL. DMSO: seedlings grown in medium with DMSO served as the control. Scale bar corresponds to 1 cm.

We next investigated the functional consequences of the observed TADs. We evaluated the genomic features contained in the TADs and found a group of TADs constructed by *SAURs*, one of which comprised 13 350–14 220 kb on chromosome 12 was presented in the original heatmap and O/E heatmap (Figure 5a). This TAD with a length of ~242 kb constructed by 25 *SAURs* present in all three organs (Figure 5a; Figure S5a). Comparison of the Hi‐C heatmaps from dark and light conditions showed that this *SAURs*‐containing TAD was dynamic under light across the three organs; notably, in the hypocotyl, this domain was more condensed under light than in the dark (Figure 5a; Figure S5a). Additionally, we found that in the hypocotyl, this *SAURs*‐containing TAD was covered with a high amount of elongating RNA polymerase II (as indicated by RNAPII Ser2P) under darkness, while RNAPII Ser2P peaks decreased under light (Figure 5a). Thus, we further measured the enrichment of RNAPII Ser2P over the entire *SAURs* gene body and found that, under darkness, RNAPII Ser2P was increased and concentrated near the *SAURs* transcription start site (TSS), while under light, RNAPII Ser2P was decreased and distributed (Figure 5b). These data suggested that elongating polymerase promotes the transcription of *SAURs* under light by coordinating with the increased chromatin interactions within TAD.

We next investigated the characteristics of *SAURs* throughout the genome and found that the soybean genome encodes 244 *SAURs* genes, which could be classified into two categories according to their distribution in the genome. The first group is comprised of 162 (~67%) *SAURs* that exist as clusters of diverse sizes (‘clustered‐*SAURs*’) and the second is comprised of 77 (~33%) *SAURs* genes that are individually distributed across the genome (‘individual *SAURs*’). We found that RNAPII Ser2P was enriched across the gene bodies of protein‐coding genes and individual SAURs in light compared to darkness (Figure S5b), which differed from the distribution of RNAPII Ser2P across clustered‐*SAURs* (Figure 5b). Interestingly, clustered‐*SAURs* were down‐regulated in the apical hook and hypocotyl with increasing illumination time (light exposure for 1, 6 and 24 h) but were upregulated under constant light. We did not observe this co‐expression pattern among clustered‐*SAURs* in the cotyledon (Figure 5c). However, individual *SAUR*s’ response to light displayed more complex patterns across the three tissues, which differed from that of the clustered‐*SAURs* (Figure S5c). Together, our results suggest that light promotes growth of the cotyledon, apical hook and hypocotyl by differentially regulating the expression of *SAURs*.

To verify the role of RNAPII in *SAURs* expression and seedling development, we applied a widely used inhibitor, flavopiridol (FVP) (Chao *et al*., 2000; Chao and Price, 2001) and germinated the soybean seeds under darkness and light for 4 days. Consequently, P‐TEFb‐mediated phosphorylation of serine 2 on the RNA polymerase II C‐terminal domain was selectively blocked by FVP, by which disrupted transcription elongation and affected the growth of the *Arabidopsis* seedlings (Chen *et al*., 2019). Our results clearly showed that development of the 4‐d‐old seedlings was disrupted by FVP. More specifically, both hypocotyl elongation and root development were strongly inhibited in plants treated with FVP compared to those treated with DMSO (dimethyl sulfoxide) (Figure 5d). Taken together, our data suggest that inhibition of transcription elongation by FVP affects the cell elongation of hypocotyl during seedling development.

**Figure 6:**
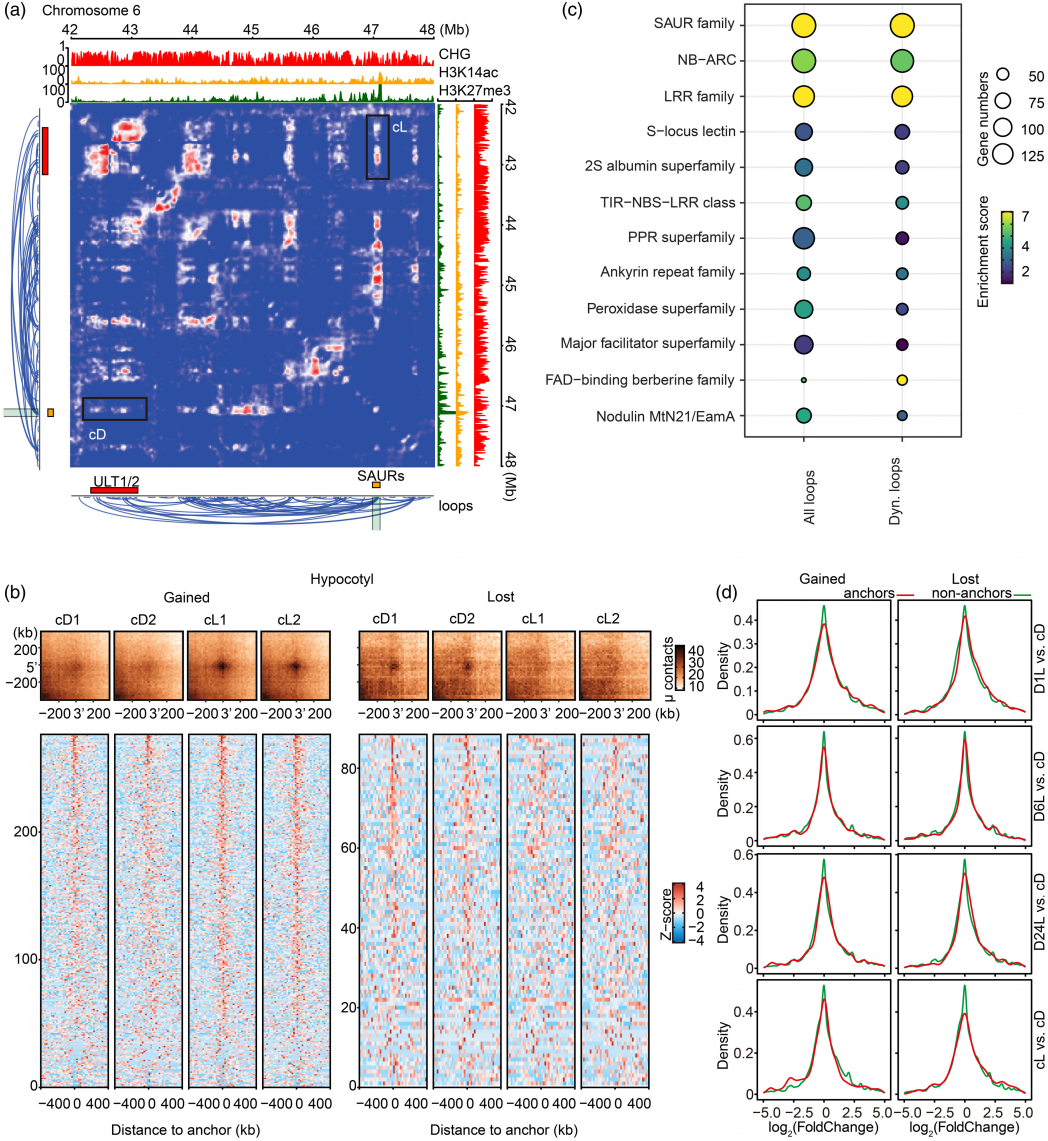
*SAURs* are involved in the dynamics of long‐range chromatin interactions. (a) Hi‐C map at 42–48 Mb on chromosome 12 showing the dynamic chromatin contacts of a *SAURs* cluster (red) and *ULT1/2* (orange) under cD and cL. cD: constant darkness. cL: constant light. Matrix resolution is 5 kb. CHG: DNA methylation in the context of CHG. H3K14ac: acetylation of the DNA packaging protein histone H3. H3K27me3: trimethylation of lysine 27 on histone 3. Black rectangle: the long‐range chromatin interactions formed by a *SAURs* cluster and *ULT1/2*. (b) Aggregate Peak Analysis (APA) of the dynamic long‐range chromatin interactions, including gained and lost chromatin loops in hypocotyl. All (intra‐chromosomal) interactions in the 20‐kb resolution matrix were averaged. Gained: The loops with more interactions under light compared to that under dark. Lost: The loops with fewer interactions under light compared to that under dark. cD1 and cD2: two replications of constant darkness treatment. cL1 and cL2: two replications of constant light treatment. (c) Gene enrichment analysis for all loops and the dynamic loops between dark and light conditions. Shapes represent the gene number. The colour of the shape represents the enrichment score. Dyn. Loops: Dynamic loops. *SAUR* family: *SAUR*‐like auxin‐responsive family. NB‐ARC: NB‐ARC domain‐containing disease resistance. LRR family: Disease resistance family protein/LRR family. S‐locus lectin: S‐locus lectin protein kinase family. 2S albumin superfamily: Seed storage 2S albumin superfamily. TIR‐NBS‐LRR class: Disease resistance protein (TIR‐NBS‐LRR class), putative. PPR superfamily: Pentatricopeptide repeat (PPR) superfamily. Nodulin MtN21/EamA: Nodulin MtN21/EamA‐like transporter family. (d) Distribution of gene expression changes at the different time points of illumination in hypocotyl. Genes located at the loop anchors are coloured red, while the genes out of the chromatin loops are marked green.

In addition to the chromatin interactions observed within the *SAURs* cluster, we also found that clustered *SAURs* at 46 984 580‐47 109 819 strongly interacted with the genomic region containing *ULTRAPETALA1/2 (ULT1/2)* genes, which are reported to encode trxG (Trithorax‐group)‐like function proteins in plants (Monfared *et al*., 2013). It is also reported that ULT1 and ULT2 co‐localize to regulate developmental genes in a tissue‐specific manner (Monfared *et al*., 2013). In our study, We demonstrated that these long‐range chromatin contacts between *ULT1/2* and an H3K27me3‐marked *SAURs* cluster became more condensed under light (Figure 6a). Globally, we identified thousands of dynamic loops among the three organs (cotyledon: 1081, hook: 653 and hypocotyl: 788) during the transition from dark to light, which included both gained loops (indicated by intense peaks) and the lost loops (disappearing peaks) under light (Figure 6b; Figure S6a,b). *SAURs* appear to be the most enriched gene family at the anchors of the dynamic loops (Figure 6c). Among three tissues, genes within dynamic chromatin loops tend to have higher expression variance than those outside the dynamic chromatin loops (Figure 6d; Figure S6c,d). When we specifically measured epigenetic features across dynamic interactions in hypocotyl, we found the accumulation of both H3K9me2 and H3K27me3 in gained loops and the enrichment of H3K27me3 became higher in gained loops under light, whereas the deposition of H3K9me2 on lost loops tended to be stable against the light stimulation (Figure S6e). This observation suggested that the H3K27me3 might be mediated with the construction of the large (≥ 10 kb) chromatin structures induced by light. Our findings also expand on earlier reports suggesting that chromatin loops bring together key genomic elements to modulate gene expression in various plants, such as *Arabidopsis*, rice and maize (Huang *et al*., 2021; Louwers *et al*., 2009; Zhao *et al*., 2019).

## Discussion

In our study, we probed soybean morphology during the de‐etiolation process and described the spatial genome structure within these tissues at different scales. We found that chromatin organization plays a critical role in light responses during seeding development, with observing the more condensed chromatin under light in the cotyledon, apical hook and hypocotyl. Moreover, we are the first to achieve yielding epigenetic features by CUT&tag in soybean, the resulting data demonstrated that the histone marks and transcription sign are closely related to the chromatin activity is consistent with the findings of other studies (Deng *et al*., 2023; Domb *et al*., 2022; Liu *et al*., 2016; Sun *et al*., 2024; Wang *et al*., 2015, 2021). Over each chromosome, the enrichment of H3K4me3, RNAPII Ser2P, H3K27ac and H3K27me3 was displayed on the euchromatin defined as the active chromatin region, which was in contrast to the repressive pericentromeric accumulation of H3K9me2. In line with the conclusions of previous research in soybean, A/B compartments, TADs and chromatin loops are the typical spatial characteristics of chromatin conformation (Ni *et al*., 2023; Wang *et al*., 2021). However, we further identified a type of new domain in soybean, termed sub‐compartments. Chromatin within sub‐compartments switched between active and repressive states in response to light and this switch was closely related to transcriptional activity. In particular, we found that light‐responsive transcriptional activity of light‐related genes was mediated by TADs and long‐range chromatin interactions.

Interestingly, the genomes of all three soybean organs exhibited TADs, which is a typical chromatin architecture found in animals; in plants, their role remains under debate. TADs have been divided into two groups in mammals: self‐interacting domains formed by the cohesin complex at the CTCF‐binding site via DNA loop extrusion (Vietri Rudan *et al*., 2015; Zhu and Wang, 2019) and CTCF‐independent TADs identified in vertebrates, which highly correlate with histone modifications (Bintu *et al*., 2018; Szabo *et al*., 2020). Despite exhibiting physical domains at the kilobase scale, *Drosophila* differs from mammals in that *Drosophila* domain boundaries are independent of active and repressive histone modifications, which are located at regions with high gene density and transcriptional activity, a finding supported by the observed clustering of RNAPII and transcription factors at these sites (Hou *et al*., 2012). In plants, the absence of CTCF and the lack of understanding of the mechanisms underlying chromatin folding made it controversial to define such interacting domains; thus, these domains are labelled TADs or compartment domains (CDs) in plants (Dong *et al*., 2017, 2020; Yin *et al*., 2023). A previous study in rice found enrichment of the TCP‐binding motif at the border of TADs and further genetic experiments conducted in *Marchantia* showed that the chromatin interactions within the TCP1‐rich TADs and TCP1‐bound TAD borders were minimally affected by the absence of *TCP1* (Karaaslan *et al*., 2020; Liu *et al*., 2017), suggesting that the formation and maintenance of TADs might be independent of *TCP1*. These observations make precise determination of the location of TADs and the proteins that directly mediate the establishment of chromatin folding difficult, which largely limits further study of 3D genome organization and its function in plants. Inspired by our data showing that the TADs identified in three soybean organs were altered under light, we further investigated protein binding of these TADs and found that a group of TADs that were dynamic under changes in light was enriched for RNAPII Ser2P in a distinct pattern. Our work suggests that RNAPII might function as a mediator of chromatin contact construction and modification. This conclusion is also expanded to the research of plants, it is suggested that the gene–gene chromatin interactions are positively correlated with the binding of RNAPII among the highly transcribed genes in *Arabidopsis* (Liu *et al*., 2016). The recently published study using Micro‐C‐XL approach further indicated that the RNAPII involves the chromatin interaction at single‐gene level by comparing the occupancy of RNAPII CTD on *nrpb2‐3‐* and FVP‐affected genes. Additionally, this research also successfully built the Micro‐C‐XL heatmap of soybean at 800‐bp resolution, which provided superior data over our study (Sun *et al*., 2024).

However, an important remaining question is how chromatin folding mediated by RNAPII regulates the transcription landscape. Increasing evidence suggests a complicated association between transcription and genome 3D organization. In terms of self‐interacting domains, RNAPII has been described as an active mark of TADs in rice and was also found to be involved in gene activation by participating in promoter‐promoter chromatin interactions (Zhao *et al*., 2019). Similarly, RNAPII is essential not only for TAD formation but also for the organization of enhancer‐promoter interactions during the cell cycle in human cells (Zhang *et al*., 2021, 2023). In murine embryonic stem cells, depletion of RNAPII compromised most short‐range chromatin interactions within 100 kb involving the genes and their cis‐regulatory elements (Jiang *et al*., 2020). RNAPII, transcription factors and other key mediators cooperatively associate with chromatin via the phase‐separation condensate to regulate transcription (Boehning *et al*., 2018; Boija *et al*., 2018; Cho *et al*., 2018). Combined with our findings showing that light‐induced tissue‐specific expression of *SAURs* is mediated by RNAPII‐associated TADs, it is tempting to speculate that the chromatin structure, such as TADs and loops, participates in the regulation of gene expression by RNAPII. Yet, we still lack clear direct evidence of the mechanism of regulation by RNAPII, which may involve specific proteins and sequences.

We also found that the transcription elongation of clustered‐*SAURs* under light was clearly distinct from that in darkness. For decades, we have understood the significance of transcription elongation in gene expression during responses to environmental stimuli. For example, at the *HSP70* (heat shock protein 70) locus in *Drosophila*, RNAPII is recruited, poised and paused at the promoter region and specifically resumes transcription after pausing following heat shock (Rougvie and Lis, 1988). For non‐expressed genes, however, RNAPII is found only at the promoter (Margaritis and Holstege, 2008). Our findings predict that the mechanism of transcription elongation of light‐related genes in response to light stimuli will differ from that under darkness. Furthermore, treatment with FVP has been shown to significantly affect the development of root tips by inhibiting cell division in the shoot apical meristem (Cheng *et al*., 2021). Our study found that both root development and hypocotyl elongation under light were disrupted by FVP, indicating that, besides cell division, FVP may also affect cell elongation induced by light. However, we offer no explanation of the mechanisms of FVP inhibition of hypocotyl elongation. Additional molecular biology experiments are needed to address this mechanism.

## Conclusions

In this study, we revealed 3D genome architecture as a potential layer in the regulation of light‐related genes in soybean. This discovery is key for understanding how higher‐order chromatin structure mediates regulation of light‐related genes and for prediction of critical transcription factors and epigenetic regulators that may orchestrate light‐responsive chromatin reorganization.

## Methods

### Plant material and growth conditions

The soybean cultivar Williams 82 (Wm82) was used for all photomorphogenesis experiments described in this study. All seeds were sterilized by treatment with chlorine gas for 14–18 h and sowed in bottles containing B5 medium, 2% sucrose and 1% phytagel. Seeds were incubated for 4 days in a growth chamber (Percival) set at 26 °C and 126.9 μmol/m^2^/s Photosynthetic Photon Flux Density for white light treatment. Following incubation, the cotyledon, apical hook and hypocotyl were separately collected for RNA‐seq, CUT&tag and *in situ* Hi‐C. For the FVP treatment experiments, 15 μM FVP was applied to the B5 medium prior to germinating the seeds under dark and light.

### RNA‐seq and data analysis

Total RNA was extracted using the RNeasy Plant Mini kit (Qiagen) and DNA was removed by using RNase‐Free DNase (Qiagen) during the RNA extraction. Beijing Novogene Co. Ltd performed construction of Poly‐A RNA‐seq libraries using the NEBNext® Ultra™ RNA Library Prep Kit for Illumina® (NEB, USA). Libraries were sequenced on the Illumina NovaSeq 6000 platform to generate 150‐bp paired‐end reads. The program fastp (https://github.com/OpenGene/fastp) with the paired‐end (PE) input/output setting was used for quality control and filtering of the read pairs. Filtered reads were mapped to the *Glycine max* reference genome (Wm82.a2.v1, https://www.soybase.org/) using hisat2 (Kim *et al*., 2019) with the flags ‐hisat2‐x < hisat2‐idx > −1 <m1> −2 <m2> ‐S < hit> and annotated using TopHat2 (Kim *et al*., 2013) using the Glycine_max_v2.1 Annotation (https://ftp.ensemblgenomes.ebi.ac.uk/pub/plants/release‐57/gff3/glycine_max/). Aligned reads were sorted and compressed using Samtools (Li *et al*., 2009), which was optimized for fast random access by bamCoverage. Read counts were normalized using Reads Per Kilobase per Million mapped reads (RPKM). Furthermore, WiggleTools was employed to calculate the coverage mean of two biological replicates and featureCounts (Liao *et al*., 2014) was used to count the number of fragments assigned to each genomic feature (e.g., genes) with the argument ‐p. The differential gene expression of five treatment pairs (D1L vs. cD, D6L vs. cD, D24L vs. cD, cL vs. cD) in which each light treatment was compared with the constant dark condition was analysed using DESeq2 (Love *et al*., 2014) with strict criteria (q < 0.05 and fold change >2). The reproducibility of the two biological replicates was evaluated by PCA using the top 2000 differentially expressed genes (DEGs) ranked by decreasing expression level. Gene expression heatmaps were generated using ComplexHeatmap and all possible logical relations between groups of activated genes under light among the three tissues were displayed using Venny 2.1.0 (Venny 2.1.0 (csic.es)). GO analyses were conducted using agriGO V2.0 (http://systemsbiology.cau.edu.cn/agriGOv2/index.php) with default parameters.

### CUT&tag

CUT&tag was performed using the Hyperactive® Universal CUT&tag Assay Kit for Illumina Pro (Vazyme Biotech, TD904) following the manufacturer's protocol with some modifications. Briefly, frozen tissues were ground with liquid nitrogen and soaked in a Nuclei Permeabilization Buffer. Following filtering with a 40‐μm cell filter and 22‐μm Miracloth, the nuclei were collected and counted with a cell counter plate. The extracted nuclei (~100 000) were gently suspended in 100 μL Wash Buffer (Vazyme Biotech, TD904‐01) and incubated with Cona Beads (Vazyme Biotech, TD904‐02) for 5 min. Wash Buffer was removed using a magnetic separation rack (NEB, S1515S) and the sample was incubated with 1 μL primary antibody (H3K9me2, Abcam, ab1220; H3K4me3, Affinity, DF6935; Phospho‐Rpb1 CTD (Ser2) (E1Z3G) Rabbit mAb, CST, 13499; H3K27ac, Abcam, ab4729; H3K27me3, Diagenode, C15410195; Normal Rabbit IgG, CST, 2729) and 50 μL corresponding antibody buffer at 4 °C overnight. After removing the primary antibodies with a magnetic separator, the nuclei were incubated with a secondary antibody in 50 μL Dig Wash Buffer at room temperature for 1 h followed by three washes with Dig Wash Buffer. Concurrently, pA‐Tn5 transposase and TTBL were prepared by diluting them with Dig 300 Buffer at 1:50 and 1:4, respectively. The nuclei were incubated with pA‐Tn5 transposase at room temperature for 1 h and washed three times with Dig 300 Buffer. The nuclei were then incubated in TTBL for 1 h at 37 °C. Mixed complexes were combined with 2 μL 10% SDS and 1 μL DNA Spike and incubated at 55 °C for 10 min. The supernatant was transferred to DNA Extract Beads Pro (Vazyme Biotech, TD904‐02) and incubated for 20 min and then the beads were washed twice with 200 μL 1 × B&W buffer for 30 s. DNA was fully eluted with 15 μL ddH2O and amplified by 2 × CAM (Vazyme Biotech, TD904‐01). The amplification was purified with VAHTS DNA Clean Beads (Vazyme Biotech, #N411) and sequenced on the Illumina Novaseq platform.

### CUT&tag data analysis

Raw sequencing data from CUT&tag was collected and filtered using fastp (Chen *et al*., 2018). Bowtie 2 (Langmead and Salzberg, 2012, 2) was used to map reads to the *Glycine max* reference genome (Wm82.a2.v1, https://www.soybase.org/). After removing the PCR duplicates using MarkDuplicates (http://broadinstitute.github.io/picard/), SAMtools (Li *et al*., 2009) was used to retain uniquely mapped reads. Clean reads were then transformed to bigwig files using bamCoverage from deepTools with the following options: binSize 10, normalizeUsingRPKM and corresponding effectiveGenomeSize. Reads were normalized by input using the bamCompare with ‐operation subtract (Ramírez *et al*., 2016). For better evaluation and analysis of CUT&tag data, the blacklists that provide the problematic regions allowing for the anomalous signals in epigenomic data were excluded by cisDynet (Zhu *et al*., 2023). Data quality was checked by comparing the typical loci with the published data using Integrative Genomics Viewer (IGV) (Robinson, 2011).

### Hi‐C library construction and sequencing

The cotyledon, apical hook and hypocotyl were individually collected from seedlings that were grown in constant darkness or constant light for 4 days. Each tissue was cut into pieces and frozen at −80 °C before the experiment. Tissues were crosslinked in formaldehyde. The resulting fixed nuclei were used for construction of Hi‐C libraries by Frasergen Gene Technology Co., Ltd (Beijing, China). Tissue pieces were soaked in 2 % formaldehyde solution for 15 min followed by quenching with glycine. After nuclei filtering and collection, the chromatin was digested using the restriction enzyme MboI. Then, biotin‐14‐dCTP was used to isolate the crosslinked DNA fragments from all MboI‐digested fragments by labeling the end of the 5′ overhang of the cross‐linked DNA fragments and the fragment ends were joined. Proteins were removed using proteinase K and DNA was purified using phenol and chloroform. The non‐ligated fragments were removed from Biotin‐14‐dCTP using T4 DNA polymerase. By combining streptavidin C1 magnetic beads and biotin‐14‐dCTP, ligated fragments 200–600 bp in size were enriched and their A‐tailing was performed with Klenow fragment (exo‐). Using a ligation mix, Illumina paired‐end sequencing adapters were ligated to the DNA fragments, which were then used as the template for PCR. Hi‐C libraries were generated by 12–14 cycles of PCR and were sequenced using a BGISEQ‐2000 to generate 2 × 150 bp reads.

### Hi‐C data processing with HiC‐Pro

Hi‐C sequencing reads generated by the BGI sequencer were preprocessed by fastp. The end‐to‐end algorithm in Bowtie2 was used to align the reads to the *Glycine max* reference genome (Wm82.a2.v1, https://www.soybase.org/), which provided gapped alignments. HiC‐Pro was used to detect unmapped reads that spanned the ligation sites and process the clean read pairs (Servant *et al*., 2015). These two steps discarded reads with low mapping quality, multiple hits and singletons. Valid interactions were defined as aligned read pairs involving different restriction fragments and we observed a distribution of molecule sizes between the restriction site and each read position. Dangling ends, self‐ligations and valid pairs duplicated due to PCR were filtered out by HiC‐Pro. We generated several genome‐wide HiC contact maps with valid interactions by calculating the proper map resolutions and dividing the genome into equal sizes, as described in our previous report (Sun *et al*., 2023). Since the downstream analysis required different formats for the Hi‐C matrix, we used HiCExplorer (https://github.com/deeptools/HiCExplorer/) to transform the h5 format using hicTransform with fixed bins for contact maps.

### Hi‐C map normalization and sample comparison

Hi‐C contact maps underwent two normalization steps in HiCExplorer. First, in order to enable comparison of different Hi‐C contact matrices, hicNormalize was used to normalize all matrices to the lowest read count of the given matrices. Second, the Knight‐Ruiz (KR) matrix balancing algorithm (Knight and Ruiz, 2013) within hicCorrectMatrix was used to correct data biases. To visualize the Hi‐C contact matrix for the cotyledon, apical hook and hypocotyl in darkness and light, the maximum and minimum score values among the six matrices were extracted and used to construct the heatmaps for dark and light treatments for each tissue at 100‐kb resolution using hicPlotMatrix. To determine the effect of light on chromatin structure, hicCompareMatrices was used to calculate the log_2_ratio and hicPlotMatrix was used to build differential Hi‐C heatmaps for dark and light treatment for each tissue. The differences in genome‐wide chromatin interactions between dark and light for each tissue were quantified by an average scaling plot using the interaction decay exponent (IDE), which is a slope of the linear fit of mean contacts intensities. Then, a distance‐dependent decrease of interaction preference was calculated using hicPlotDistVsCounts in HicExplorer and was then visualized using ggplot2 (http://ggplot2.tidyverse.org).

To identify the chromatin features and changes that occurred in response to light stimuli, we used the PCA analysis function in HOMER (http://homer.ucsd.edu/homer/interactions2/index.html) to define the chromatin compartmentalization of each individual chromosome. Reads were pooled in 10‐kb bins at 15‐kb intervals to create the distance‐normalized interaction matrix using analyzeHiC. PCA analysis was performed using the runHiCpca.pl program with a 50‐kb resolution and 200‐kb window size. The orientation of resulting eigenvalues was corrected for the H3K27ac and H3K27me3 modifications. Juicebox software was used to ensure that positive PC1 values corresponded to A compartments and negative PC1 values corresponded to B compartments. To measure the change in the strength of the compartmentalization between the dark and light conditions, the genome‐wide compartment strength was calculated and visualized using GENOVA (GitHub – robinweide/GENOVA: GENome Organisation Visual Analytics) using the 100‐kb contact matrix, which calculates the score from the observed over expected (O/E) scores within the compartment interaction bins divided by the square of the O/E scores between compartments. The more defined compartment characteristics (sub‐compartments) were inferred using CALDER (GitHub – CSOgroup/CALDER), as previously described (Liu *et al*., 2021). The screenshots of typical loci were generated using Integrative Genomics Viewer (IGV). Based on the corrected matrix, TAD structures were called by calculating the TAD separation scores for a series of window sizes, which were generated by moving the 5‐kb step from the minimum window length of 15 kb to the maximum window length of 60 kb. TAD structures were displayed using hicPlotTAD. To quantify the strength of the TADs by ATA analysis, as has been done in previous studies (Flyamer *et al*., 2020; Sun *et al*., 2020, 2023), we applied coolpup.py to map the average interactions of TADs and borders with the 5‐kb‐resolution Hi‐C matrix. To create local rescaled pileups of the TADs, each TAD was padded with flanks of the same length as the TAD and then they were all rescaled to 87 × 87 pixels. The valleys of the insulation scores were calculated and averaged to quantify the TAD boundaries.

### Detection, quantification and comparison of long‐range chromatin interactions

To further identify the long‐range chromatin loops among millions of contacts, Hi‐C maps were prepared on a genome‐wide scale at 5‐, 10‐ and 20‐kb resolution in cooler format using hic2cool (https://github.com/4dn‐dcic/hic2cool) and normalized using the balance function in cooler (Abdennur and Mirny, 2020). Next, Chromosight was employed to call loops in merged cD (control) samples with a minimum distance of 100 kb and a maximum distance of 2 Mb using a loop pattern. The loop score, a metric of the similarity of the detected patterns to the loop template, was measured based on the Pearson Correlation Coefficient by (Matthey‐Doret *et al*., 2020). To identify the most precise loops, we retained only the loops with loop scores >0.35, >0.4 and >0.45 at the 5‐, 10‐, or 20‐kb resolution, respectively. We have previously observed that heterochromatin regions contained by pericentromeric and centromere regions exhibit relatively poor mapping quality and chromatin loop patterns distinct from those found on the chromosome arms (Sun *et al*., 2023). Thus, the tool pairToBed in bedtools (Quinlan and Hall, 2010) was applied to exclude the chromatin loops identified from heterochromatin regions. We captured the remaining loops derived from the contact maps at the three different resolutions by merging them into a union set with hicMergeLoops from hicExplorer (Wolff *et al*., 2018) with the option ‐lowestResolution 40 000. Juicebox was used to check the chromatin loops in .hic format (Durand *et al*., 2016). To quantify the genome‐wide loop strength in *cis*, APA analysis was also performed against all loop peaks with coolpup.py (Flyamer *et al*., 2020; Sun *et al*., 2020, 2023).

## Funding

This work was supported by the National Natural Science Foundation of China (NSFC) Key Program (32230006), the Key R&D Program of Shandong Province, China (ZR202211070163), the National Top Young Talents Program of China, and Boya Postdoctoral Program of Peking University.

## Author contributions

ZL and LS performed the bioinformatic analysis. ZL and LS compiled the figures and tables. XX performed the CUT&tag experiment. YL participated in the relavant expriments. ZL wrote the original draft. XD, HH and LS revised the manuscript. All authors read and approved the manuscript.

## Conflict of interest

The authors declare that there are no conflicts of interest.

## Supporting information


**Figure S1** Dynamics of chromatin interactions at the genome‐wide level are induced by light in the cotyledon and hook.

**Figure S2** Quality control for Hi‐C, CUT&tag and RNA‐seq data.

**Figure S3** Transcriptional activity inside the A/B sub‐compartments was associated with domain activity among three tissues.

**Figure S4** TADs were changed upon light across three organs.

**Figure S5** *SAURs* constructed‐TADs change upon the light in a tissue‐specific manner.

**Figure S6** Changes of genes expression inside the chromatin loops is mediated by light in cotyledon and hook.

**Table S1** Quality control by HiCPro.

**Table S2** Quality control of CUT&tag.

**Table S3** Specifically upregulated genes in cotyledon.

**Table S4** Specifically upregulated genes in apical hook.

**Table S5** Specifically upregulated genes in hypocotyl.

## Data Availability

The raw sequencing data generated in this study are available at the Genome Sequence Archive (GSA, https://bigd.big.ac.cn/gsa/; accession number: CRA011652).
