# Supplementary material for: Light control of three‐dimensional chromatin organization in soybean
Source: Plant Biotechnol J. 2024 May 19;22(9):2596–611. doi: 10.1111/pbi.14372 (PMC11331798; doi:10.1111/pbi.14372)
Supplement: Supplementary file 1 — Figure S1 Dynamics of chromatin interactions at the genome‐wide level are induced by light in the cotyledon and hook. [file PBI-22-2596-s003.docx]

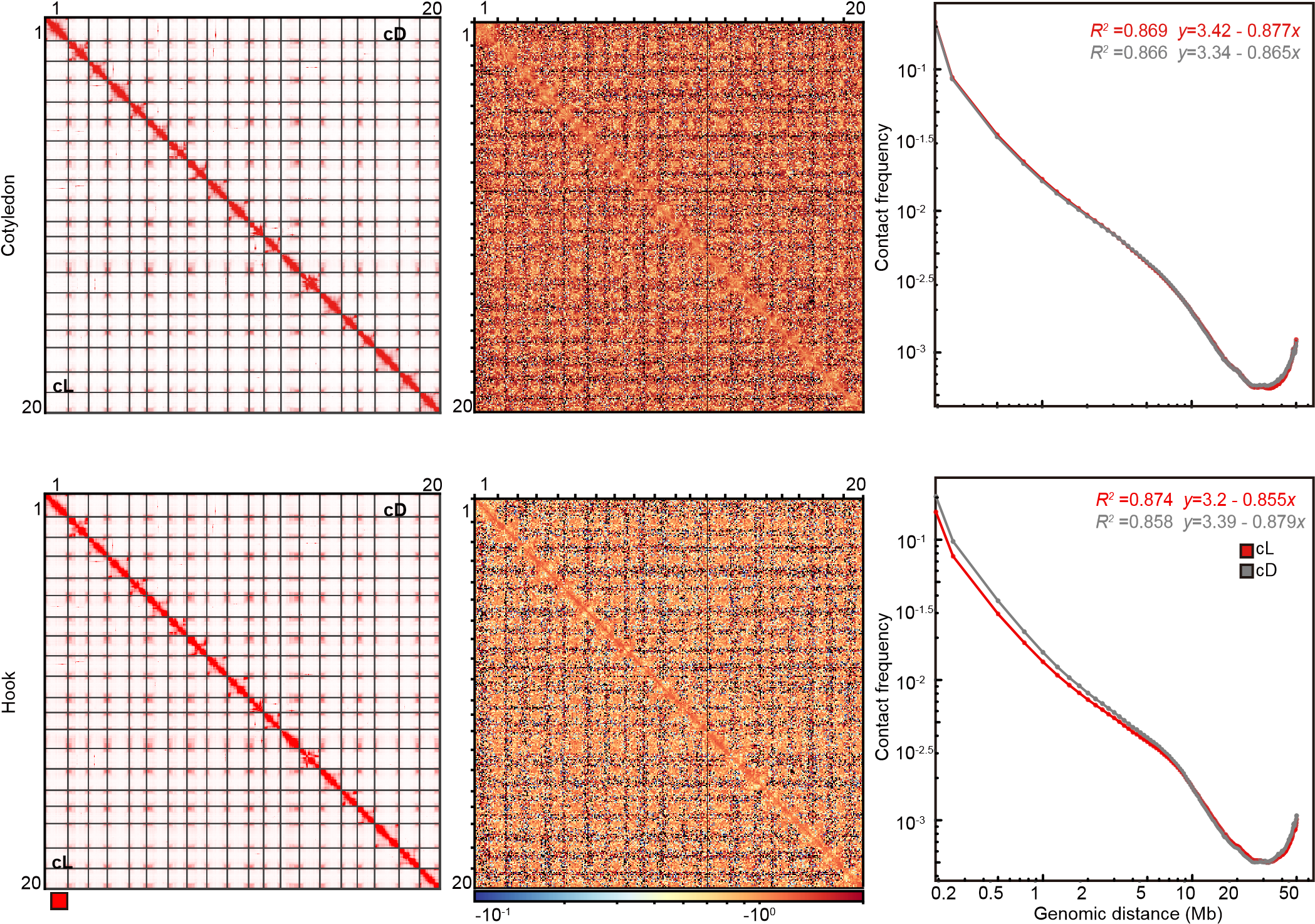


Supplementary Fig. 1

**Fig. S1 Dynamics of chromatin interactions at the genome-wide level are induced by light in the cotyledon and hook.** Shown is the genome-wide chromatin architecture of cotyledon and hook under both cD and cL as revealed by the chromatin interaction frequency at 100-kb resolution. cD: constant darkness. cL: constant light. Left: heatmap showing the chromatin interaction patterns over twenty chromosomes under continuous light (bottom left corner) and darkness (top right corner). Diagonal line values are set to zero. Middle: Heatmap showing the chromatin interactions that remain after subtracting the contacts observed under darkness from those observed under light. Right: averaged scaling plots showing the interaction frequencies with increasing genomic distance for twenty chromosomes compare the interaction decay exponents (IDEs) of darkness and light. The genomic bin size was set to 100 kb.
