# Supplementary material for: Light control of three‐dimensional chromatin organization in soybean
Source: Plant Biotechnol J. 2024 May 19;22(9):2596–611. doi: 10.1111/pbi.14372 (PMC11331798; doi:10.1111/pbi.14372)
Supplement: Supplementary file 2 — Figure S2 Quality control for Hi‐C, CUT&tag and RNA‐seq data. [file PBI-22-2596-s002.docx]

a

−60

−40

−20

0

20

40

60

−30

−20

−10

0

10

20

30

−30

−20

−10

0

10

20

30

PC2 (15.5%)

cD

cD

cD

D1L

D1L

D1L

D6L

D6L

D6L

D24L

D24L

D24L

cL

cL

cL

Hook

Hypocotyl

Cotyledon

cD

D1L

D6L

D24L

cL

0.9900

0.9925

0.9950

0.9975

1.0000

100000

40000

20000

10000

5000

SCC score

Rep1 vs. Rep2 (cD)

Rep1 vs. Rep2 (cL)

d

Bin size (bp) PC1 (62.3%)


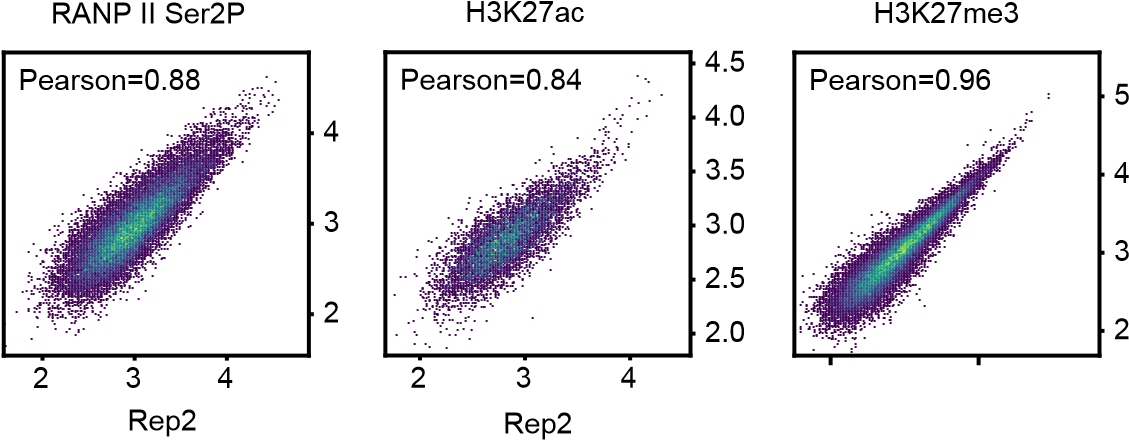
 2 4

b


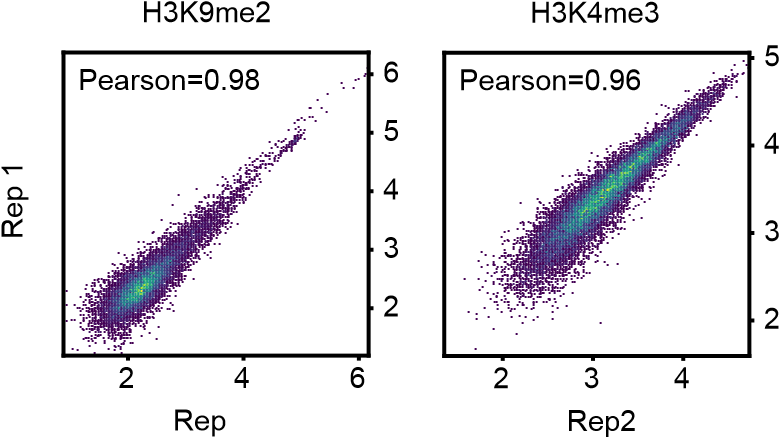


Rep2

| Chromosome 14 | | | | | | Chromosome 14 | | | | | | | |
| --- | --- | --- | --- | --- | --- | --- | --- | --- | --- | --- | --- | --- | --- |
| 22,843,893-22,913,679 (bp) | | | | | | 3412,319,799-12,354,691 (bp) | | | | | | | |
| 42  0 |  | |  |  | | 56  0 |  | | | | | | |
| 42  0 |  | | | | | 56  0 |  | | | | | | |
| 42  0 |  | | | | | 56  0 |  | | | | | | |
| 42  0 |  |  |  |  |  | 56  0 |  |  |  |  |  |  |  |
|  |  |  |  |  |  |  |  |  |  |  |  |  |  |
| 42  0 |  |  |  |  |  | 56  0 |  | | | | | | |
|  |  |  |  |  |  |  |  |  |  |  |  |  |  |
| 42  0 |  | | | | | 56  0 |  | | | | | | |
|  | | | | | |  | | | | | | | |

c

H3K9me2 Rep1 (cD)

H3K9me2 Rep2 (cD)

Input (cD)

H3K9me2 Rep1 (cL)

H3K9me2 Rep2 (cL)

Input (cL)

Gene Glyma.14G110000

| \| Chromosome 10 \| \| \| \| \| \| \| --- \| --- \| --- \| --- \| --- \| --- \| \| 42,358,409-42,376,753 (bp) \| \| \| \| \| \| \| 139  0 \|  \|  \|  \| \|  \| \|  \| \|  \|  \|  \|  \| \| 139  0 \|  \| \|  \| \| \| \| \| \| \| 139  0 \|  \| \| \| \| \| \| 139  0 \|  \| \|  \|  \|  \| \|  \| \| \| \| \| \| \| 139  0 \|  \| \|  \|  \| \| \|  \| \| \| \| \| \| \| 139  0 \|  \| \| \| \| \| \|  \| \| \| \| \| \| | \| Chromosome 8 \|  \| \| --- \| --- \| \|  \| 3,283,802-3,301,186 (bp) \| |
| --- | --- | --- | --- | --- | --- | --- | --- | --- | --- | --- | --- | --- | --- | --- | --- | --- | --- | --- | --- | --- | --- | --- | --- | --- | --- | --- | --- | --- | --- | --- | --- | --- | --- | --- | --- | --- | --- | --- | --- | --- | --- | --- | --- | --- | --- | --- | --- | --- | --- | --- | --- | --- | --- | --- | --- | --- | --- | --- | --- | --- | --- | --- | --- | --- | --- | --- | --- | --- | --- | --- | --- | --- | --- | --- | --- | --- | --- | --- | --- |
|  | \| 51  0 \|  \| \|  \|  \|  \|  \| \| --- \| --- \| --- \| --- \| --- \| --- \| --- \| \| 51  0 \|  \| \|  \|  \|  \|  \| \| 51  0 \|  \| \| \| \| \| \| \|  \| \|  \| \|  \| \|  \| \| 51  0 \|  \| \|  \| \| \| \| \| \| \| \| 51  0 \|  \| \| \| \| \| \| \|  \| \|  \| \| \| \| \| \| 51  0 \|  \| \|  \| \| \| \| \| \| \| |

H3K27me3 Rep1 (cD)

H3K27me3 Rep2 (cD)

Input (cD)

H3K27me3 Rep1 (cL)

H3K27me3 Rep2 (cL)

Input (cL)

Gene Glyma.10G190800 Glyma.10G190900 Glyma.08G041600

Supplementary Fig. 2

**Fig. S2 Quality control for Hi-C, CUT&Tag and RNA-seq data.** (a) Scatterplot summarizing the Stratum-adjusted Correlation Coefficient (SCC) values between

biological replicates of each Hi-C sample, reflecting good repeatability. (b) Correlation between two biological replicates for RNAP II Ser2P and various histone modifications under constant dark (cD). Pearson Correlation Coefficients (PCC) were calculated from the peaks generated from CUT&Tag data. (c) Integrated Genome Browser (IGV) showing high reproducibility between cD and cL for H3K9me2 and H3K27me3. Left panel showing the stable peak between cD and cL. Right panel showing the decreased peak under cL compared to cD. (d) Principal component analysis (PCA) of RNA-seq data. The shapes represent different soybean tissues. The colors of the shapes represent different growing conditions.
