# Supplementary material for: Light control of three‐dimensional chromatin organization in soybean
Source: Plant Biotechnol J. 2024 May 19;22(9):2596–611. doi: 10.1111/pbi.14372 (PMC11331798; doi:10.1111/pbi.14372)
Supplement: Supplementary file 3 — Figure S3 Transcriptional activity inside the A/B sub‐compartments was associated with domain activity among three tissues. [file PBI-22-2596-s007.docx]

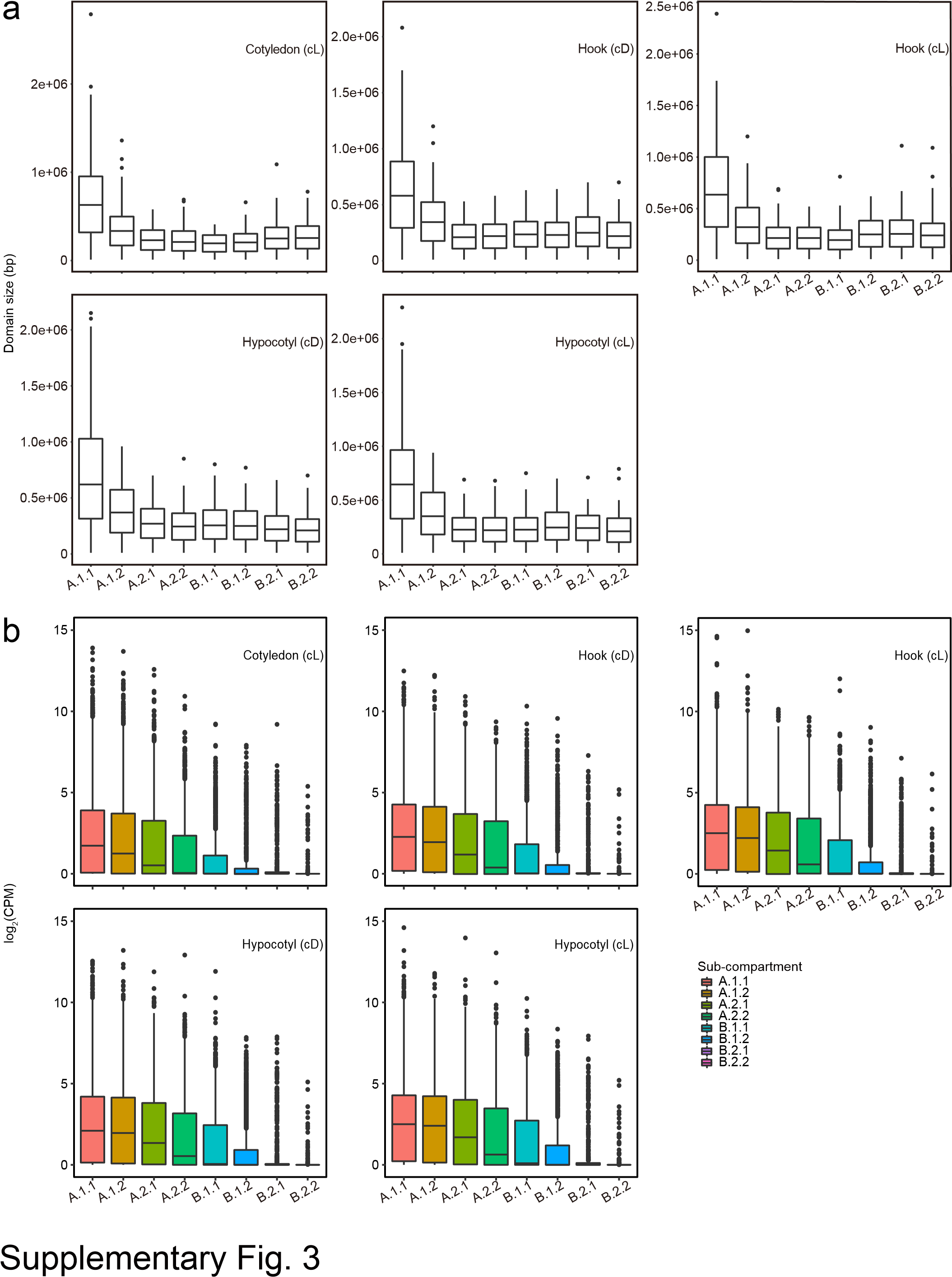


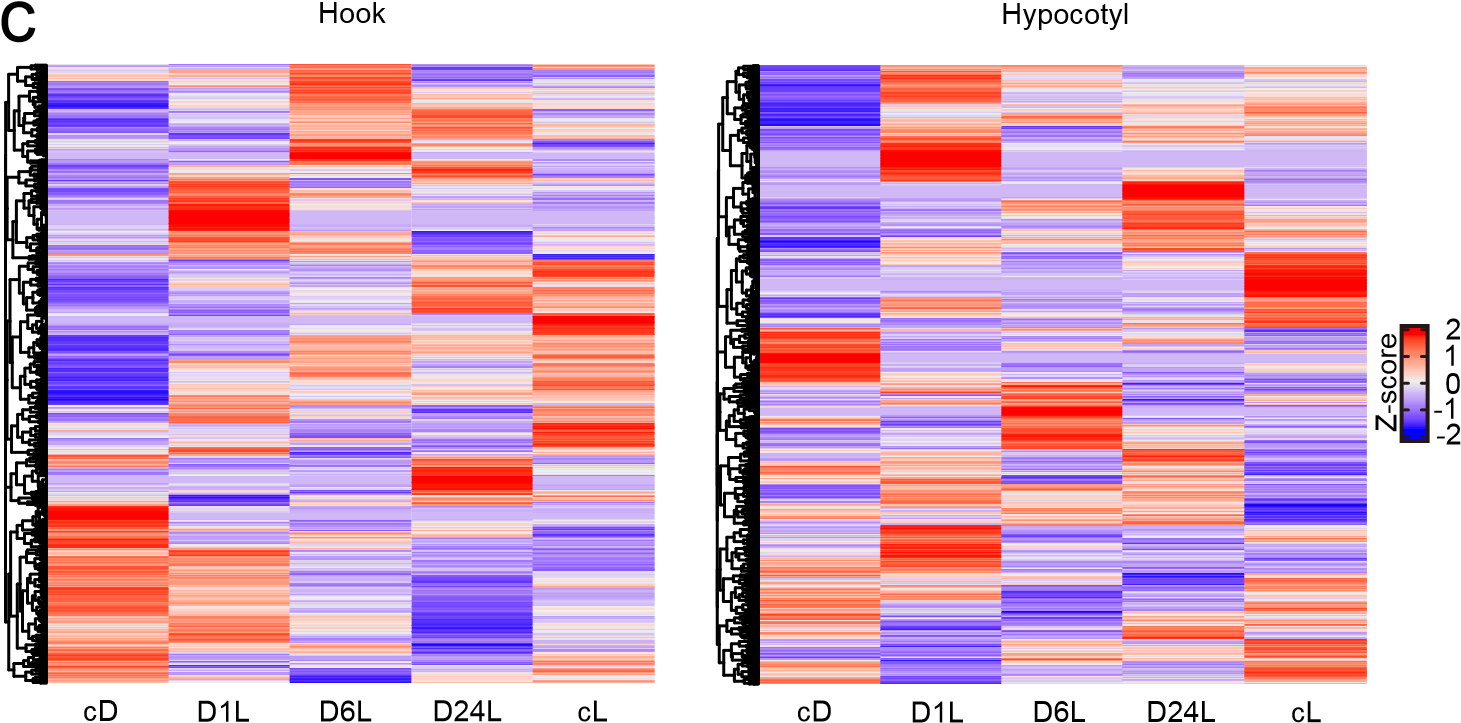


Supplementary Fig. 3

**Fig. S3 Transcriptional activity inside the A/B sub-compartments was associated with domain activity among three tissues.** (a) Domain size distribution of all types of sub-compartments in three tissues subjected to cD and cL. cD: constant darkness. cL: constant light. (b) Boxplot showing the transcription level of genes contained within each type of sub-compartment in three organs under both cD and cL. Y-axis: log_2_ (CPM). (c) Heatmap visualizing the expression of genes under the following growth conditions: constant dark (cD), dark followed by light for 1 h (D1L), dark followed by light for 6 h (D6L), dark followed by light for 24 h (D24L), and constant light (cL). The genes were selected from the B-A sub-compartments in the apical hook and hypocotyl samples. Z-scores were computed on a gene-by-gene (row-by-row) basis by subtracting the mean and then dividing by the standard deviation. Z-scores range from -2 to 2.
