# Supplementary material for: Light control of three‐dimensional chromatin organization in soybean
Source: Plant Biotechnol J. 2024 May 19;22(9):2596–611. doi: 10.1111/pbi.14372 (PMC11331798; doi:10.1111/pbi.14372)
Supplement: Supplementary file 4 — Figure S4 TADs were changed upon light across three organs. [file PBI-22-2596-s010.docx]

a


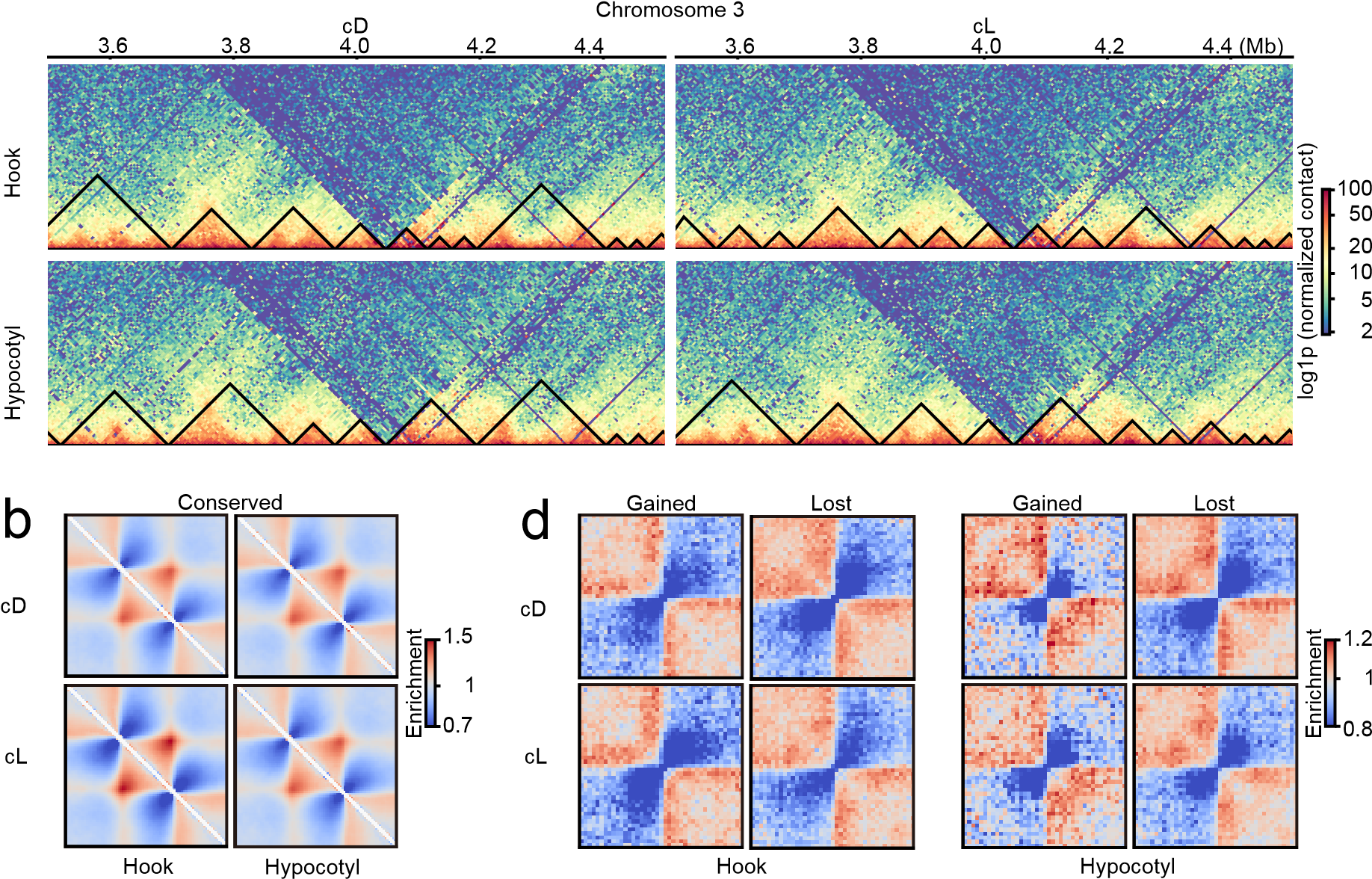


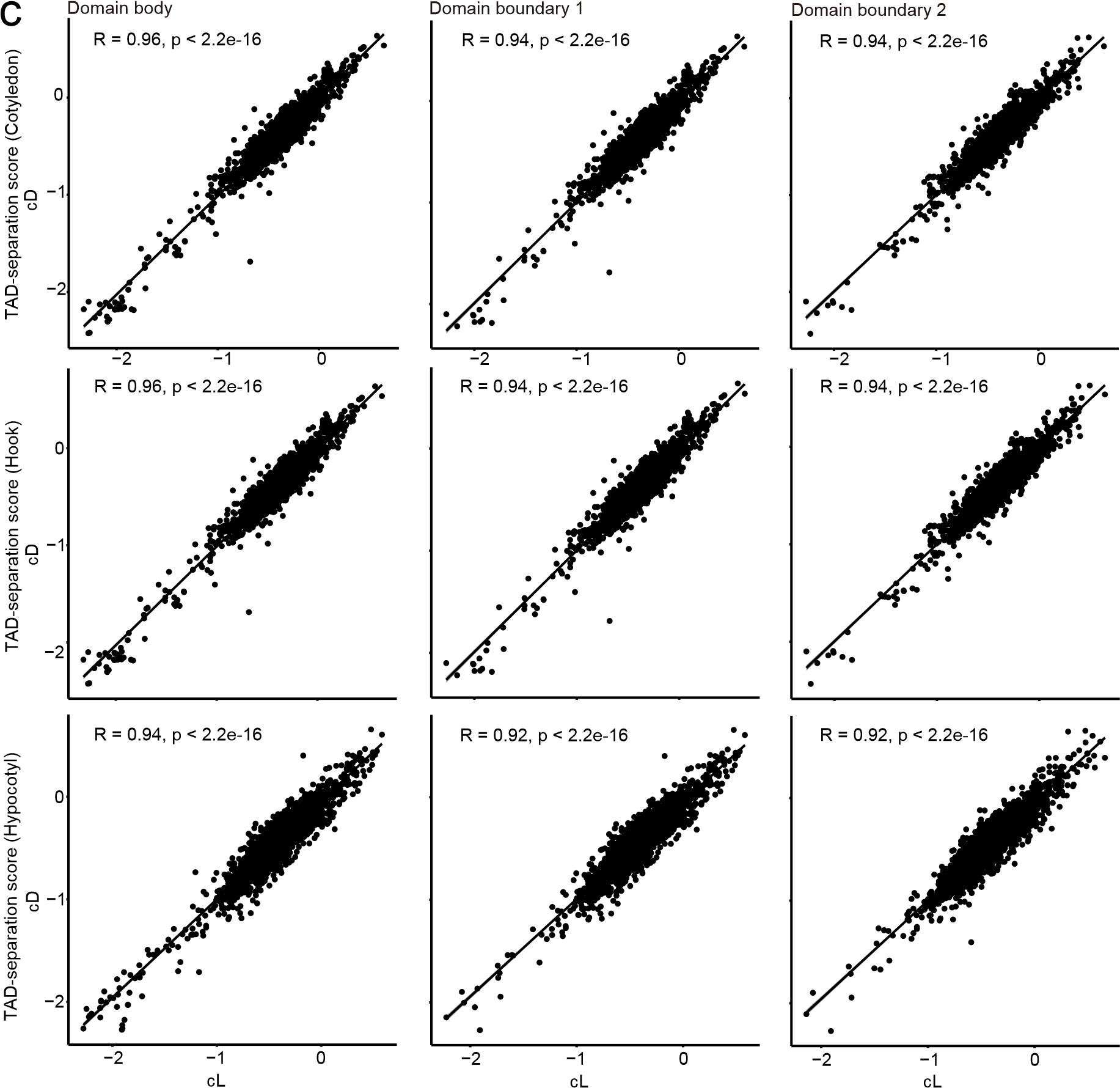


Supplementary Fig. 4

Gained Lost

f

Detection of light stimulus

Sugar mediated signaling path

way

Response to et

h

ylene

Maintenance of cell n

umber

Enrichment score

5

10

15


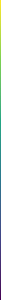


0.4

0.6

0.8

1.0

-

log

10

(FDR)

Cotyledon

Hook

Hypocotyl

Gained

Lost

Cotyledon

Hook

Hypocotyl

Photosynthesis, light reaction

Cellular response to light stimulus

Shoot system morphogenesis

Leaf development

Ethylene−activated signaling pathway

Response to carbohydrate

−0.4

−0.2

0

0.2

−100

kb

0

100

kb

−100

kb

0

100

kb

e

Hook

Hypocotyl

−0.4

−0.2

0

0.2

cD

cL

TADs insulation score

Histone modification

Positive regulation of histone modification

Regulation of protein ubiquitination

Protein autophosphorylation

Secondary metabolite biosynthetic process

Symbiotic interaction

# Supplementary Fig. 4

**Fig. S4 TADs were changed upon light across three organs.** (a) Snapshot of Hi-C heatmap showing the shape of the TAD over a 1-Mb genomic region (3.5-4.5 Mb) on chromosome 3 in the apical hook and hypocotyl under both cD and cL conditions. A matrix with a 5-kb resolution was used for TAD calling. The normalized heatmap was transformed by log1p. cD: constant darkness. cL: constant light. (b) Aggregate TAD analysis (ATA) of conserved TADs domains in both complete darkness and light. 5-kb-resolution Hi-C matrices were used for the pileups analysis of the TADs. Enrichment bar: average insulation values relative to random background, shown from highest to lowest. (c) Scatterplot showing the relationship between interactions in the conserved TADs under both darkness and light. The p-value was generated using a two-sided Pearson Correlation Test. Left: comparison of interactions in conserved TADs domains under dark and light. Middle and right: comparison of interactions in the left and right boundaries of conserved TADs under dark and light. (d) Aggregate TAD analysis (ATA) for the boundaries of dynamic TADs. Gained TADs and lost TADs were separately defined as those with higher and lower insulation scores under light compared to those under dark. 5-kb-resolution Hi-C matrices were used for pileups analysis of the TADs. Average insulation was quantified by dividing the signal into two red squares (top left and bottom right corners) by the signal in the blue squares (top right and bottom left corners). (e) Profiling of TAD insolation scores across the dynamic borders of both cotyledon and hook. Blue: dynamic TADs observed under continuous dark. Green: dynamic TADs observed under constant light. The borders of gained and lost domains are aligned and marked as ‘0’. (f) Gene Ontology (GO) analysis of the genes contained within dynamic TADs. The X-axis shows different tissues, and Yaxis shows the GO terms describing biological processes. The color of the circles represents the value of -log_10_ (FDR). The size of the circles represents the enrichment score of the corresponding GO term. The enrichment score was calculated as (number of input genes in GO terms / number of input genes) / (number of genes in GO terms/ number of total genes).
