# Supplementary material for: Light control of three‐dimensional chromatin organization in soybean
Source: Plant Biotechnol J. 2024 May 19;22(9):2596–611. doi: 10.1111/pbi.14372 (PMC11331798; doi:10.1111/pbi.14372)
Supplement: Supplementary file 5 — Figure S5 SAURs constructed‐TADs change upon the light in a tissue‐specific manner. [file PBI-22-2596-s004.docx]

Cotyledon Hook

a


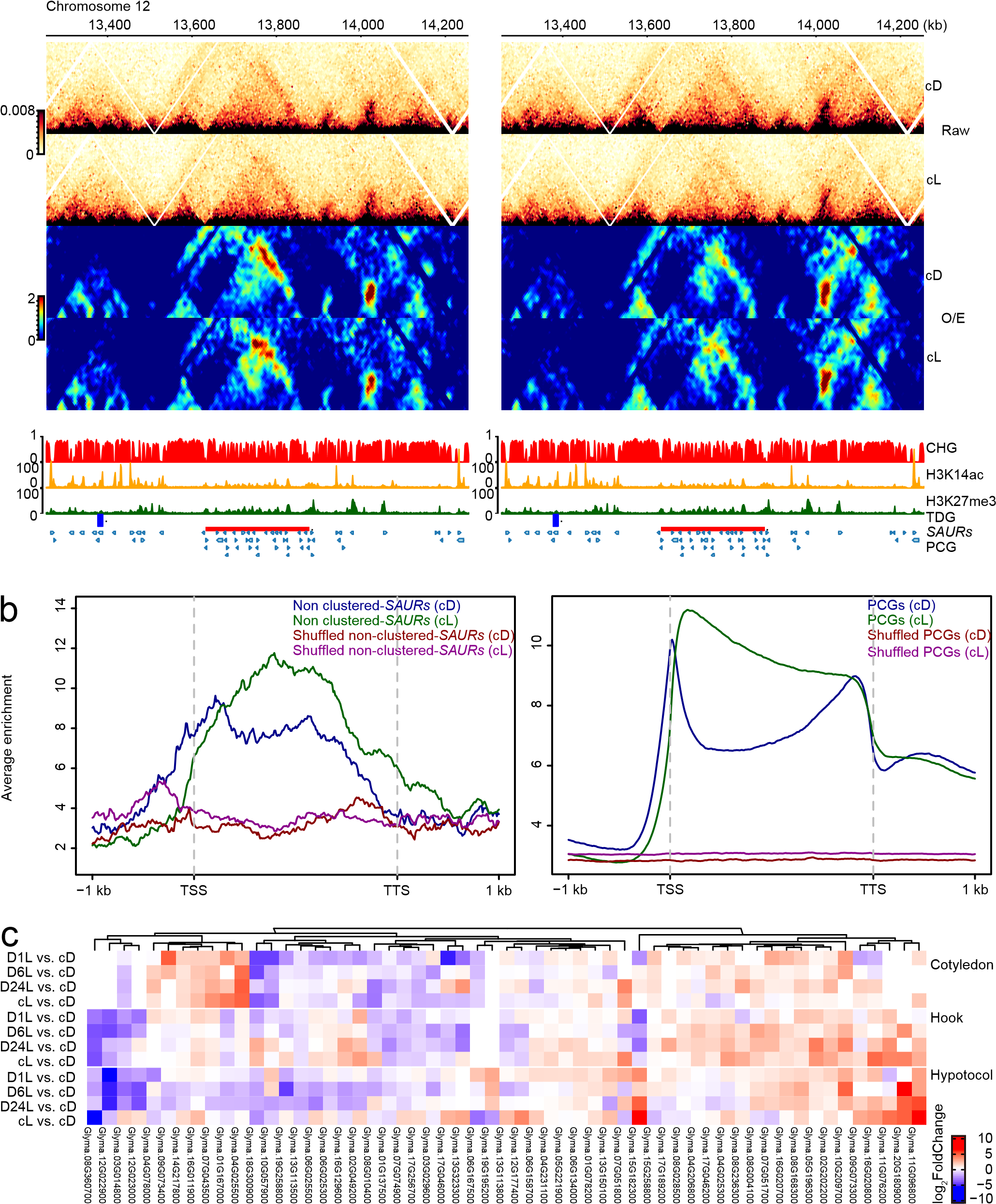


# Supplementary Fig. 5

**Figure. S5 *SAURs* constructed-TADs change upon the light in a tissue-specific manner.** (a) Snapshot of TAD identified at 13,300-14,250 kb on chromosome 12 in the cotyledon and apical hook under cD and cL. cD: constant darkness. cL: constant light. A matrix at 5-kb resolution was used for TAD calling. CHG (red track): DNA methylation in the context of CHG. H3K14ac (yellow track): acetylation of the DNA packaging protein histone H3. H3K27me3 (green track): trimethylation of lysine 27 on histone 3. TDG (blue bar): Tandemly Duplicated Gene. *SAURs* (red bar): Small Auxin-Upregulated genes. The distributions of CHG methylation, H3K14ac, and H3K27me3 were extracted from publicly available data (Wang *et al.*, 2021). (b) Distribution of RNAPII Ser2P across the gene body of ‘non-clustered *SAURs’* and all protein-coding genes (PCGs). The average enrichment of RNAPII Ser2P over the ‘non-clustered *SAURs’* or PCGs under darkness and light is shown by blue and green curves, respectively. Red and pink curves in the plots indicate background signal, which was calculated by distributing the ‘non-clustered *SAURs’* or PCGs randomly throughout the genome for both the dark and light conditions. The flanking regions at the left side of the transcription start site (TSS) and at the right side of the transcription terminal site (TTS) are 1 kb in length. (c) Expression heatmap of nonclustered *SAURs* in the cotyledon, apical hook, and hypocotyl. The expression levels in the D1L, D6L, D24L, and cL samples were normalized using the cD sample and are shown as log_2_ FoldChange, which ranges from -10 to 10. The annotated names on the bottom of columns are the gene IDs of the soybean *SAURs*.
