# Supplementary material for: Light control of three‐dimensional chromatin organization in soybean
Source: Plant Biotechnol J. 2024 May 19;22(9):2596–611. doi: 10.1111/pbi.14372 (PMC11331798; doi:10.1111/pbi.14372)
Supplement: Supplementary file 6 — Figure S6 Changes of genes expression inside the chromatin loops is mediated by light in cotyledon and hook. [file PBI-22-2596-s008.docx]

a


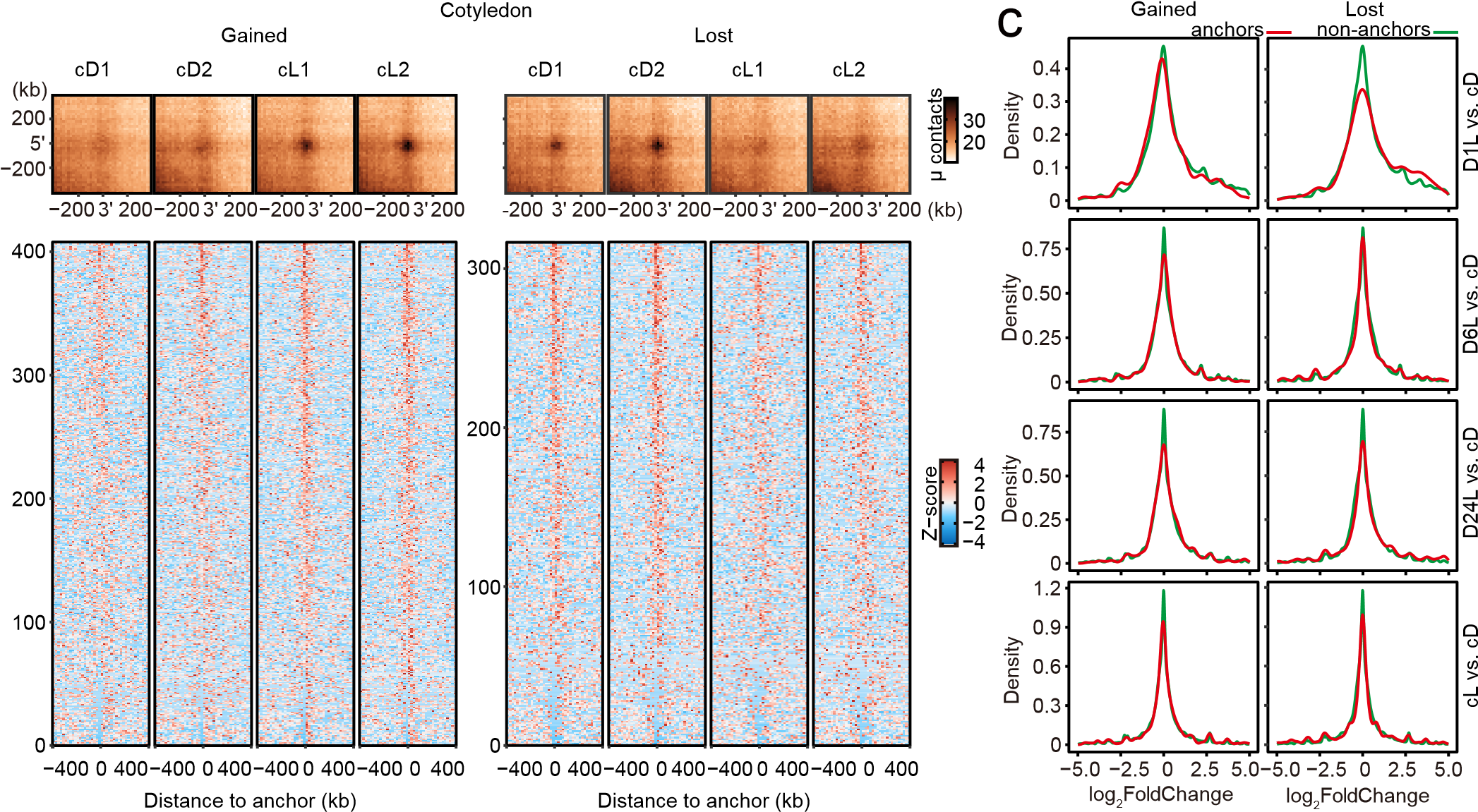


b


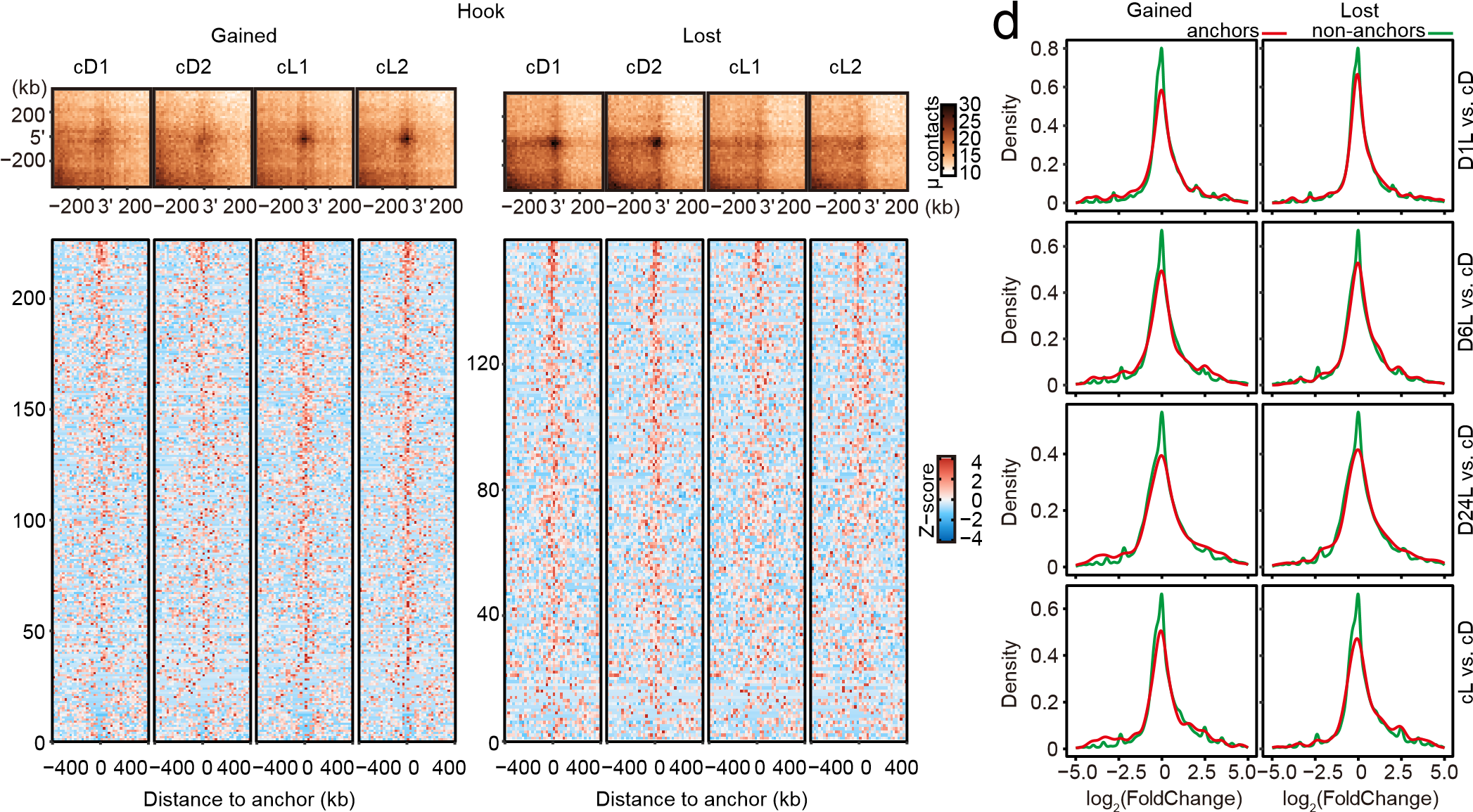


# Supplementary Fig. 6

Shuffled gained loops (cL)

1.0

1.5

2.0

2.5

3.0

3.5

4.0

2.0

3.0

4.0

5.0

6.0

0.5

1.0

1.5

2.0

2.5

3.0

3.5

kb

−40

left

right

40

kb

2.0

3.0

4.0

5.0

kb

−40

left

right

40

kb

H3K9me2

H3K27me3

e

Average enrichment

Average enrichment

Shuffled gained loops (cD)

Gained loops (cL)

Gained loops (cD)

Shuffled lost loops (cL)

Shuffled lost loops (cD)

Lost loops (cL)

Lost loops (cD)

# Supplementary Fig. 6

**Figure. S6 Changes of genes expression inside the chromatin loops is mediated by light in cotyledon and hook.** (a, b) Aggregate Peak Analysis (APA) of the dynamic longrange chromatin interactions, including gained and lost chromatin loops in cotyledon and hook, respectively. All (intra-chromosomal) interactions in the 20-kb resolution matrix were averaged. Gained: The loops with more interactions under light compared to that under dark. Lost: The loops with fewer interactions under light compared to that under dark. cD1 and cD2: two replications of constant darkness treatment. cL1 and cL2: two replications of constant light treatment. (c, d) Distribution of gene expression changes at the different time points of illumination in cotyledon and hook, individually. Genes located at the loop anchors are colored by red, while the genes out of the chromatin loops are marked by green. (e)

The accumulation of H3K9me2 and H3K27me3 across dynamic loops under cD and cL.

The region from “left” to “right” represents loop anchors with.
